# Enhanced Fatty Acid Photodecarboxylation over Bimetallic Au–Pd Core–Shell Nanoparticles Deposited on TiO_2_

**DOI:** 10.1021/acscatal.3c03793

**Published:** 2023-11-08

**Authors:** Huiru Yang, Liang Tian, Abdessamad Grirrane, Alberto García-Baldoví, Jiajun Hu, German Sastre, Changwei Hu, Hermenegildo García

**Affiliations:** † Key Laboratory of Green Chemistry and Technology, Ministry of Education, College of Chemistry, Sichuan University, Chengdu, Sichuan 610064, P. R. China; ‡ Instituto Universitario de Tecnología Química, Consejo Superior de Investigaciones Científicas, Universitat Politecnica de Valencia, 46022 Valencia, Spain

**Keywords:** photodecarboxylation, fatty acid into biofuels, Au−Pd, core−shell, TiO_2_

## Abstract

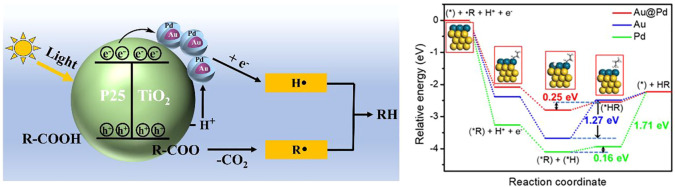

Photodecarboxylation of biomass-derived fatty acids to alkanes offers significant potential to obtain hydrocarbons and economic benefits due to the mild conditions and high activity. Herein, the photodecarboxylation of hexanoic acid into alkanes using TiO_2_-supported monometallic Au or Pd and bimetallic Au–Pd catalysts is reported. It was found that bimetallic Au–Pd catalysts, featuring a core–shell structure evidenced by EDX-mapping and element line profile, show better photocatalytic performance, achieving 94.7% conversion of hexanoic acid and nearly 100% selectivity to pentane under UV–vis irradiation in the absence of H_2_ than the monometallic Au analogue. This remarkable enhancement in activity compared to its TiO_2_ supported monometallic Au or Pd analogues can be attributed to the synergistic effect between Au and Pd within the nanostructured Au(core)-Pd(shell) alloy for achieving more efficient charge-separation efficiency upon visible light excitation. This photocatalyst exhibits a wide scope converting multiple fatty acids into hydrocarbons. Moreover, it can even photocatalyze the conversion of raw bio-oils into alkanes directly. No obvious activity loss was observed during the reusability tests, demonstrating the good stability of the present catalyst. Density functional theory (DFT) calculations indicate that oxidation of carboxylates on TiO_2_ leads to alkyl radicals that become bound to metal nanoparticles. The superior catalytic performance of Au(core)-Pd(shell)/TiO_2_ is derived from the weaker adsorption for H on the alloy and the lower hydrogen evolution reaction overpotential. Our research can result in an efficient bio-oil upgrading, resulting in the synthesis of biofuels from biomass under mild conditions.

## 1 Introduction

Biomass-derived oils, such as inedible vegetable oils (e.g., jatropha oil) or wasted cooking oils, are suitable feedstocks for the production of biofuels. These oils contain large amounts of carboxylic acids and esters.^1−3^ Decarboxylation of fatty acids derived from biomass results in hydrocarbons that can be used as synthetic fuels with zero CO_2_ footprint.^4,5^ The process has attracted much current interest to provide synthetic gasoline, jet-fuel and gas oil.^6−9^ Among the various decarboxylation processes, photocatalytic decarboxylation is very convenient and appealing due to the mild conditions, high selectivity and the possibility to use natural solar light as the ultimate energy source.^7,10,11^ Catalytic thermal fatty acid hydrogenation usually requires harsh reaction conditions to occur (*T* ≥ 250 °C, *P* ≥ 2 MPa H_2_).^12−14^

Carboxylic acid photodecarboxylation requires the development of efficient photocatalysts, and considerable research has been conducted in this area trying to find more suitable materials.^7,8,15^ In a semiconducting photocatalyst, photon absorption promotes electrons from the valence band to the conduction band, generating an electron hole in the valence band. This elementary process generates a transient charge separated state with negative electrons (e^–^) and positive holes (h^+^) pairs.^16^ The photon energy has to be equal to or higher than the bandgap of the semiconductor so that light can be effectively absorbed by the photocatalyst. TiO_2_, having a bandgap of about 3.4 eV, absorbs photons of wavelength shorter than 380 nm, but other semiconductors with narrower bandgap can be excited by visible light.^17,18^ TiO_2_ possesses several advantages, including high activity under UV irradiation, high chemical and photochemical stability against photocorrosion, high relative abundance, low toxicity, and large scale affordability. These advantages have motivated the interest for TiO_2_ in photocatalytic processes such as solar fuels production,^19,20^ environmental remediation,^21,22^ and photodecarboxylation.^7,15^

The photocatalytic activity of TiO_2_ is known to increase with deposition on its surface of metal and metal oxide nanoparticles, acting as cocatalysts.^23,24^ Although there is ample evidence that these metal nanoparticles can increase the photocatalytic activity of TiO_2_, the current data is mostly limited to monometallic nanoparticles, bimetallic nanoparticles having been considerably much less studied.^25,26^ Bimetallic nanoparticles, particularly those exhibiting unique core–shell structures, have aroused enormous interest because of their fascinating optical, electronic, sensing, and improved catalytic properties. These distinctive physicochemical and catalytic properties evolved in bimetallic nanoparticles derive from the new electronic states of the alloy which are different from individual metals.^27−30^ Furthermore, besides adaptation of the d band energy, the lattice strain created between the core and shell region as well as the tuning of the electron density at the shell also contribute to the enhanced catalytic properties of core–shell nanoparticles.^31^ Considering the numerous advantages of TiO_2_ as decarboxylation photocatalyst and the fact that the catalytic capability of core–shell metal nanoparticles remain mostly unexplored, it appears of interest to study the activity of a core–shell bimetallic catalyst deposited on TiO_2_ for the photodecarboxylation of fatty acids to obtain hydrocarbons.

In a recent publication, we have reported the photocatalytic decarboxylation activity of a series of nickel supported TiO_2_ photocatalysts for the conversion of octanoic acid into heptane and tetradecane.^15^ An octanoic acid conversion of 25% with a selectivity to heptane of about 70% was achieved at 3 h irradiation with a 300 W Xe lamp under 0.2 bar of hydrogen pressure, there being room for further improvement. Continuing with this line of research, it would be important to develop other TiO_2_ photocatalysts, showing an enhanced activity and selectivity. Herein, we report the photocatalytic activity of core–shell Au–Pd alloy nanoparticles supported on P25 TiO_2_. The results show that performance of TiO_2_ having core (Au)-shell (Pd) alloy nanoparticles exhibit a synergy in comparison with analogous TiO_2_ materials of the individual metals, giving an almost complete decarboxylation selectivity at total carboxylic acid conversion in the absence of hydrogen gas in the system. The present catalyst is not only effective in converting model carboxylic acids but also shows suitability for the conversion of crude bio-oils. This catalytic activity has been explained by DFT calculations to understand the role of the metals in the system.

## 2 Experimental Section

### 2.1 Chemicals

All chemicals were used directly as provided commercially without additional purification. Milli Q water was obtained using an IQ 7000 purifying system. P25 TiO_2_, HAuCl_4_·3H_2_O, PdCl_2_, acetone (99.9%, CH_3_COCH_3_), sodium hydroxide (reagent grade, 97%, powder, NaOH), 1-phenylethanol (97%), hexanoic acid (99%), pentane (99%), lauric acid (97%), palmitic acid (99%), stearic acid (98%), oleic acid (97%), undecane (99%), pentadecane (99%), heptadecane (99%), *p*-xylene (99.5%, as internal GC standard), different solvents including dodecane (99%), PhMe (99%), THF (99%), DMF (99%) and dichloroethane (99%) were purchased from Sigma-Aldrich. Jatropha oil was purchased from the Shenyu company in Yunnan Province, China. Wasted cooking oil and wasted hot-pot oil were collected from Sichuan Province, China.

### 2.2 Preparation of Catalysts

**Scheme 1:**
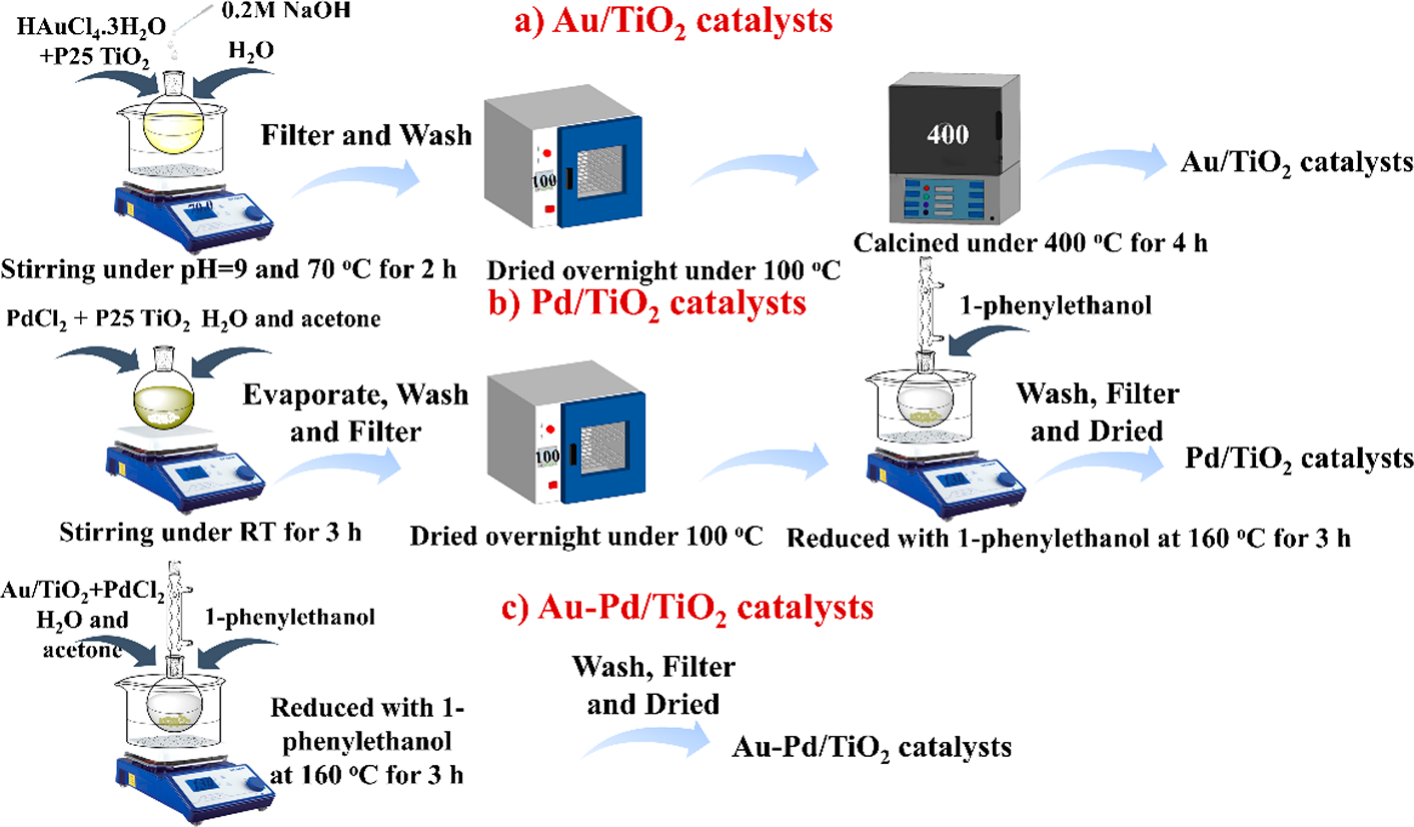
Illustration of the Preparation Procedure of TiO_2_-Based Photocatalysts. (a) Au/TiO_2_, (b) Pd/TiO_2_ and (c) Au–Pd/TiO_2_

A series of Au, Pd and Au–Pd bimetallic nanoparticles supported on P25 TiO_2_ were prepared by impregnation P25 TiO_2_ with HAuCl_4_.3H_2_O and/or PdCl_2_ under basic condition for Au/TiO_2_ or in water and acetone mixture for Pd/TiO_2_. After impregnation, the solid was dried in an oven at 100 °C and finally submitted to calcination at 400 °C (Au/TiO_2_) or reduced by treatment with 1-phenylethanol at reflux temperature (160 °C) for those samples containing Pd.^32^ The preparation procedure is illustrated in Scheme 1. Au–Pd bimetallic catalysts with different Au/Pd atomic ratios were prepared by adding in all cases the same amount of HAuCl_4_·3H_2_O, but a different amount of PdCl_2_.

For comparison, a AuPd/TiO_2_ alloy catalyst in which Au and Pd are randomly distributed in the nanoparticle was also prepared by the coimpregnation method, at an Au and Pd loading of 1.5 and 0.8 wt %, respectively. After impregnation and calcination, the sample was reduced at 400 °C for 2 h under a H_2_ flow (30 mL/min). The obtained catalyst was denoted as a 1.5Au0.8Pd alloy/TiO_2_.

### 2.3 Catalyst Characterization

Transmission electronic microscopy (TEM), EDX-Mapping and EDX element line profile were conducted at Philips CM300 FEG electron microscope with an accelerating voltage of 200 kV. The samples were first dispersed in ethanol and then treated with ultrasound (440 W) for 15 min, to obtain a homogeneous suspension. A drop of this suspension was deposited on a copper grid coated with carbon films. XRD was recorded by using a Shimadzu XRD-7000 diffractometer with CuKα radiation. The XRD patterns were recorded from 10 to 80° at a scanning speed of 10°·min^–1^. The actual loadings of Au and Pd were determined by ICP-AES using a Varian 715-ES instrument. Diffuse reflectance UV/vis spectra were recorded on a Varian Cary 5000 spectrophotometer in the wavelength range 200–800 nm. The samples were also analyzed by XPS (SPECS spectrometer with monochromated Al radiation). XPS data were calibrated taking the C 1s at 284.5 eV using Casa XPS software. The experimental high-resolution XPS peaks were fitted by using XPSPEAK1 software. The in situ IR spectra reported here were taken by BRUKER VERTEX 70 FT-IR spectrometer equipped with a high sensitivity MCT detector at a resolution of 4 cm^–1^ in the range of 1000–4000 cm^–1^ with a cell holder having KBr windows. Propionic acid was chosen as the probe molecule for IR spectroscopy measurements due to its higher vapor pressure. The detailed experimental procedure is as follows: the samples were first treated with an initial pretreatment by heating them to 150 °C under vacuum to remove adsorbed water or gases. Subsequently, the sample was cooled to room temperature. Then, propionic acid was adsorbed on the catalyst surface from the vapor phase. Following this step, the catalyst was immediately subjected to irradiation under UV–visible light.

### 2.4 Theoretical Basis

Periodic DFT calculations were conducted using the Cambridge Serial Total Energy Package (CASTEP) module^33^ with the exchange-correlation functional described by Perdew–Burke–Ernzerhof within the generalized gradient approximation (GGA-PBE).^34^ Tkatchenko and Scheffler (TS) dispersion corrections scheme^35^ was incorporated along with the exchange and correlation functional to improve the structural and vibrational properties. The anatase TiO_2_ (101) surface was presented by a three-layer slab. A 3 × 2 supercell along the [010] and [101] directions with a vacuum layer of 20 Å perpendicular to the surface was used. The system contains 130 atoms, and only the Γ point was included for sampling of the Brillouin zone. A self-consistent field method (tolerance 5.0 × 10^–7^ eV/atom) was employed in conjunction with plane-wave basis sets with a cutoff energy of 460 eV in reciprocal space. All structures are geometry-optimized until energy is converged to 5.0 × 10^–6^ eV/atom, maximum force to 0.01 eV/Å and maximum displacement to 5.0 × 10^–4^ Å. In order to avoid metal layers becoming corrugated due to geometry optimization leading toward stable nanoparticle shapes indicated above, we have constrained all the metal layers and focused on the geometry optimization into different adsorbates considered. The adsorption energy of species on the catalyst surface was calculated as *E*_ads_ = *E*_total_ – *E*_A_ – *E*_sur_, where *E*_total_ represents the total energy of the catalytic surface with an adsorbed molecule, and *E*_A_ and *E*_sur_ are the energies of isolated adsorbate molecule and the clean surface, respectively. The energy of an isolated molecule (*E*_A_) was computed by placing it in the same lattice box (about 10.2 × 11.3 × 29.3 Å^3^). The energy of the free radical is computed by a spin-polarized calculation with one unpaired electron (spin: 1).

### 2.5 Analysis of Products

The products were detected by GC with an HP-5 column. The detection program was set from 50 to 280 °C with a heating rate of 10 °C· min^–1^. The conversion, products yield and selectivity of each component were calculated by the following eqs 1–3:
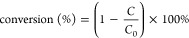
1where *C*_0_ and *C* represent the contents of substrate in the reactant and product, respectively.
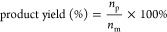
2where *n*_p_ and *n*_m_ represent the moles of product and added substrate, respectively.
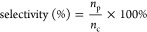
3where *n*_p_ and *n*_c_ represent the mole of product and converted substrate, respectively.

The initial reaction rates based on the metal amount were calculated by using eq 4:
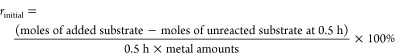
4

## 3 Results and Discussion

### 3.1 Characterizations of the Catalyst

The metal loadings of the samples prepared in the present work are provided in Table S1 of the Supporting Information. In general, the Au loading was fairly constant about 1.5 wt %, while Pd percentage increased from 0 to about 5 wt %.

**Figure 1:**
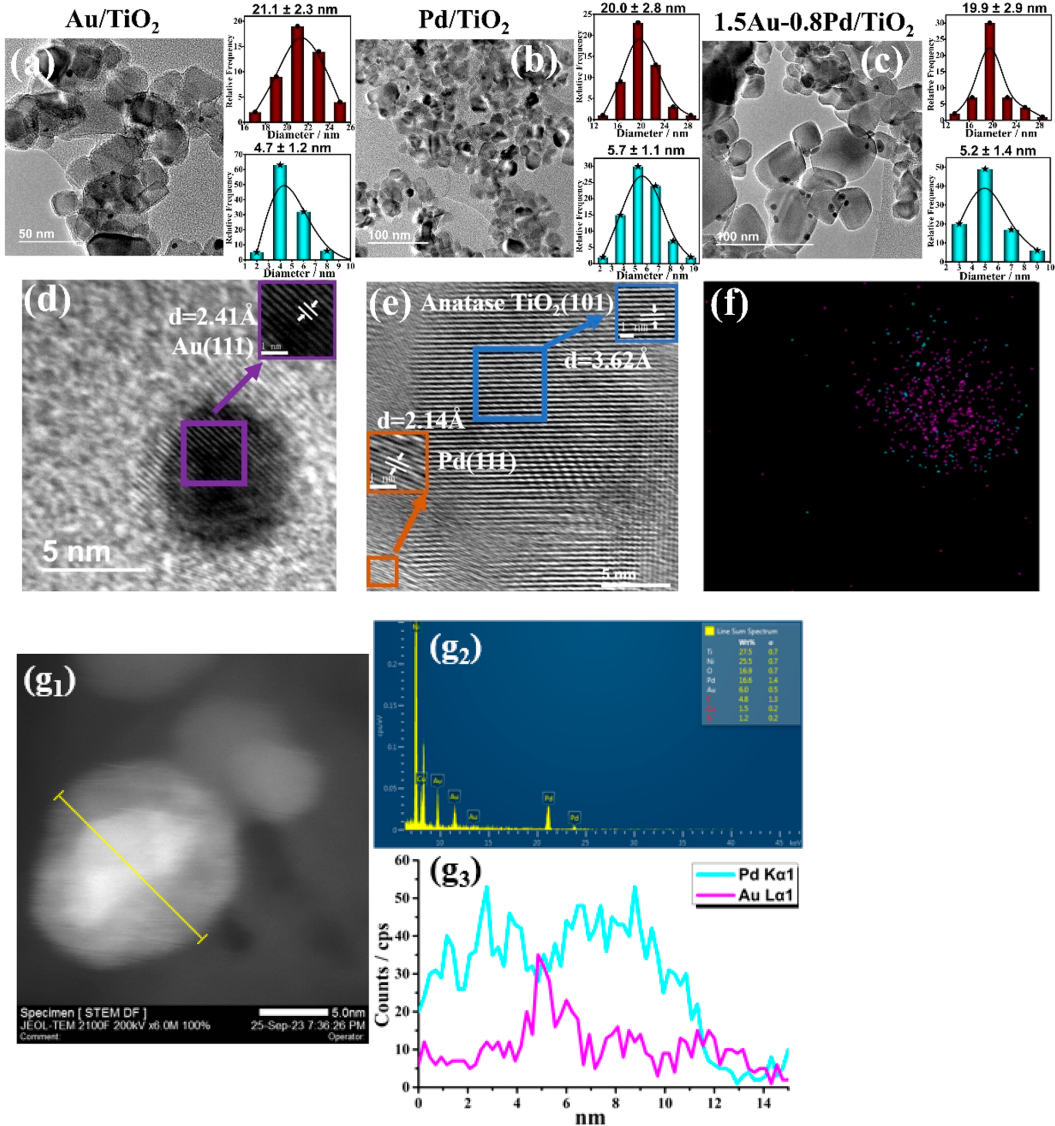
TEM images with particle size distributions (brown columns represent TiO_2_, and blue columns represent metal particles) of (a) Au/TiO_2_, (b) Pd/TiO_2_ and (c) 1.5Au-0.8Pd/TiO_2_. High resolution TEM images of (d) Au/TiO_2_ and (e) Pd/TiO_2_. The insets indicate the lattice fringe distance. EDX-mappings of Au and Pd elements of (f) 1.5Au-0.8Pd/TiO_2_ catalyst. (g_1_-g_3_) STEM images, STEM-EDX elemental profile and EDX elemental line scanning profile of 1.5Au-0.8Pd/TiO_2_ catalyst. Note that Au is located mostly in the central part of the nanoparticle.

The presence of the metal nanoparticles on TiO_2_ was determined by TEM. High-resolution images show that P25 is predominately constituted by anatase phase with less abundant rutile domains (seen in Figure S1),^36^ which is also confirmed by the XRD patterns commented later. The average particle size of TiO_2_ was in all cases about 20 nm (Figure 1). The presence of Au and Pd is observed in these images by the presence of darker, higher contrast, and smaller particles with average particle sizes about 5 nm. Figure 1d,e present selected high-resolution TEM images of some of the samples prepared in the present study. In the case of Au/TiO_2_ or Pd/TiO_2_, the interplanar distances (2.41 and 2.14 Å) of the metal nanoparticles measured by high-resolution TEM indicate that they correspond in both cases to the (111) facet of metallic Au or Pd.

Interestingly, for the 1.5Au-0.8Pd/TiO_2_ sample, elemental mapping of Au and Pd at nanometric resolution indicates that Pd is preferentially located at the external part of the Au–Pd nanoparticle, therefore having a structure of a core (Au)/shell (Pd) nanoparticle. The EDX elemental line profile (Figure 1g_1_-g_3_) across the nanoparticles further confirm the core–shell structure features of 1.5Au-0.8Pd/TiO_2_ catalyst, which would determine the electron density of the external Pd atoms and may create lattice strain. Both factors can result in a synergistic effect between these two metals. The elemental EDX-mapping of other metal/TiO_2_ photocatalysts are also provided in Figure S2 of Supporting Information. As shown in Figure S3, peaks located at 2 theta = 25.3, 36.9, 37.8, 38.6, 48.0, 53.9, 55.1, and 62.7° are ascribed to anatase TiO_2_ (PDF#21-1272), while those at 27.4, 36.1, 41.2, and 56.6° are ascribed to rutile TiO_2_ (PDF#21-1276). The anatase/rutile composition of TiO_2_ is in accordance with the images taken by high resolution TEM. No obvious peaks corresponding to metallic Au or Pd are found on these samples, a fact that is probably due to the low metal content and the small sizes of the Au or Pd nanoparticles.

**Figure 2:**
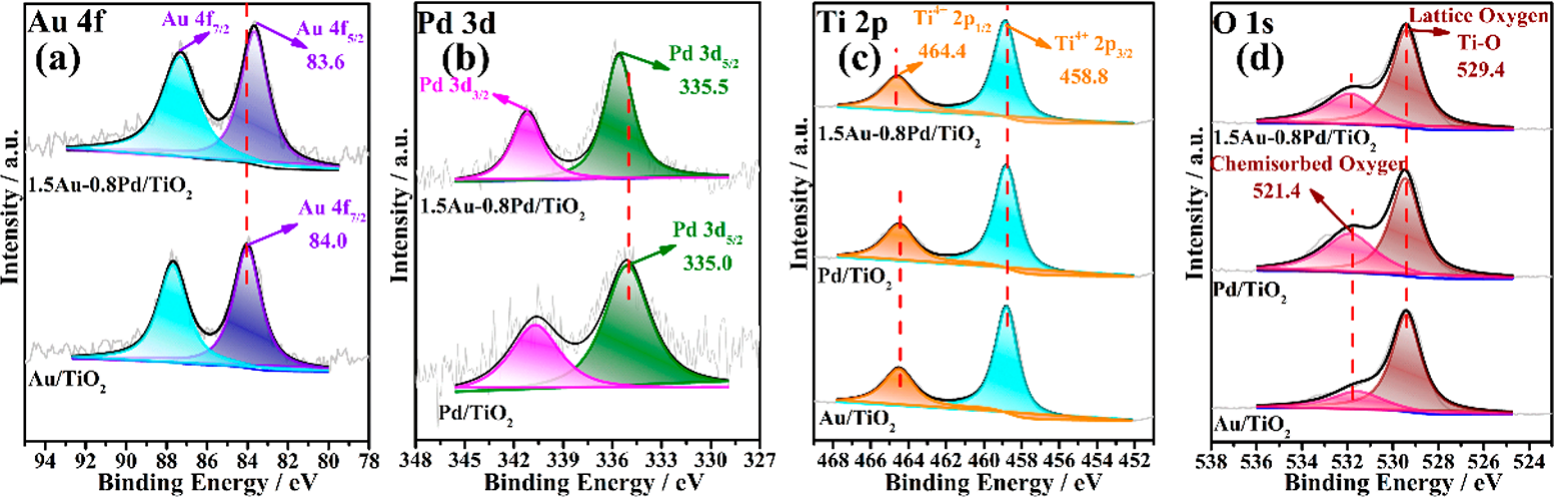
Experimental high resolution XP spectra and the corresponding deconvolution to individual components for: (a) Au 4f, (b) Pd 3d (c) Ti 2p and (d) O 1s XPS spectra of Au/TiO_2_, Pd/TiO_2_ and 1.5Au-0.8Pd/TiO_2_.

The surface chemical states of each element on the different catalysts were evaluated by XPS. Figure 2 presents the experimental XPS peaks for each element as well as their best deconvolutions to individual components. The Au 4f peaks were deconvoluted into two peaks including Au 4f _5/2_ with a binding energy around 84.0 eV and Au 4f _3/2_ near 87.6 eV, both corresponding to the metallic Au state.^37−40^ For the XPS Pd 3d spectra, the bands were also deconvoluted into two peaks corresponding to the spin–orbit splitting of the Pd (3d _3/2_) and Pd (3d _5/2_) lines (Δ = 5.26 eV), appearing at a binding energy around 335.0 and 340.3 eV, respectively.^40,41^ Compared with the monometallic catalysts, the binding energy of Au 4f _5/2_ shifts to lower energy (from 84.0 to 83.6 eV), while Pd 3d _5/2_ shifts to higher energy (from 335.0 to 335.5 eV) for the bimetallic 1.5Au-0.8Pd/TiO_2_ catalyst, indicating electron density transfer from Pd to Au.^22,42^ The presence of a strong electronic effect between Au and Pd metals in a bimetallic 1.5Au-0.8Pd/TiO_2_ catalyst can significantly improve the capability to capture electrons. The XPS Ti 2p peaks were also deconvoluted, and the peaks with binding energy values at 458.8 eV are ascribed to Ti^4+^.^43−45^ No component corresponding to Ti^3+^ was found, indicating that Ti^4+^ is the only state of Ti on the surface of these catalysts. For the O 1s spectra, they were deconvoluted into two peaks centered at 529.4 and 521.4 eV, which are ascribed to the lattice oxygen for Ti–O and chemisorbed oxygen such as −OH, respectively.^46−48^ No significant differences in binding energy are observed for both Ti 2p and O 1s between monometallic and bimetallic catalysts.

**Figure 3:**
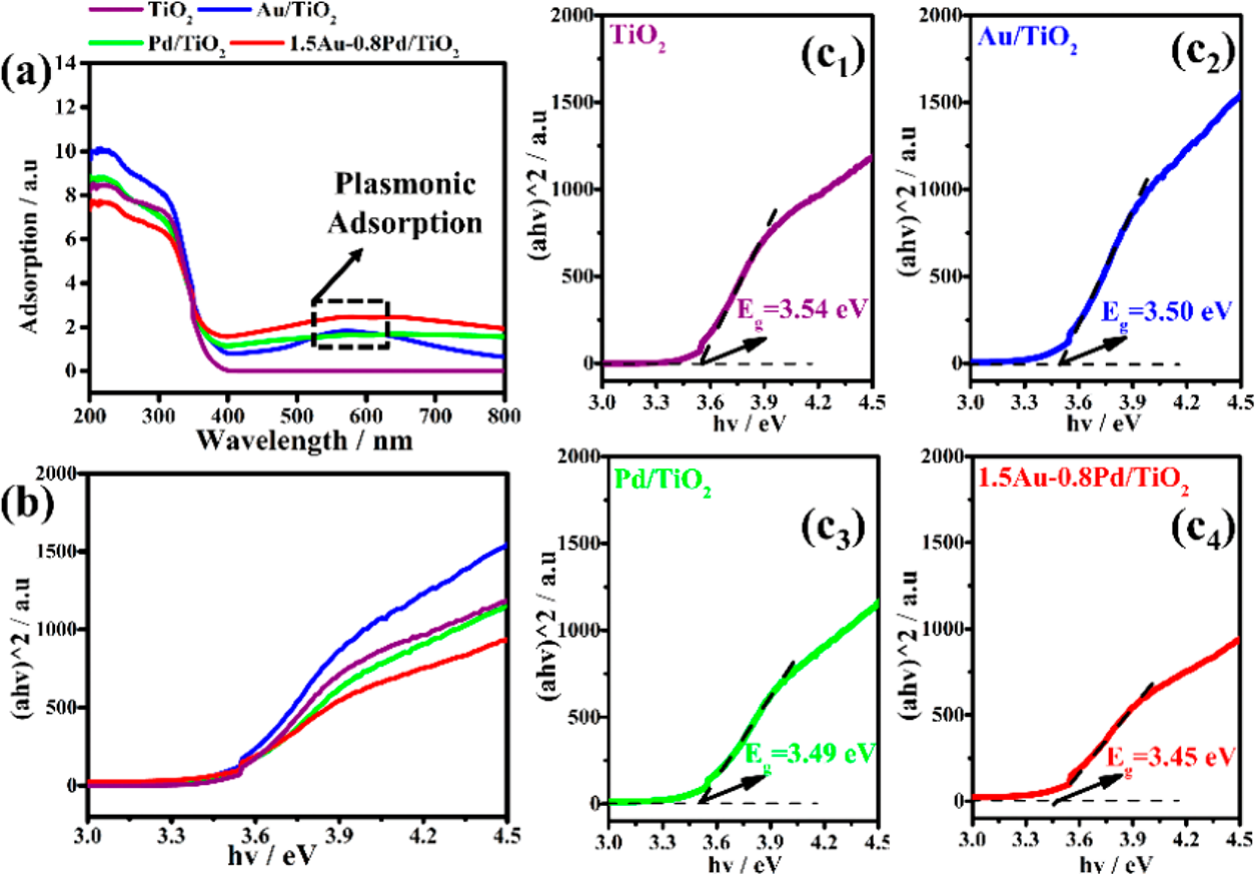
(a) UV–vis spectra of the as-prepared samples as indicated by the color codes, (b) Tauc plots of (αh*v*)^2^ versus photon energy and (c_1_-c_4_) bandgap of the samples obtained by magnification of plot (b).

Diffuse reflectance UV–vis spectroscopy is a useful tool for evaluating light absorption and electronic characteristics of photocatalytic materials. Compared with the pure P25 TiO_2_, the maxima observed in the visible range at 500–600 nm can be ascribed to the localized surface plasmon resonance (LSPR) arising from the supported Au and Pd metals (Figure 3a).^49−51^ The bandgaps were calculated using eq (5): α*hv*^1/*n*^ = A(*hv* – Eg) (5), where α is the absorption coefficient, *A* is a constant, *h* is Planck constant, Eg is the band energy, *v* is the incident light frequency, and *n* is associated with the type of semiconductor.^52^ TiO_2_ is regarded as an indirect semiconductor, wherein *n* = 2. The Tauc plots used to estimate the optical bandgaps are presented in Figure 3 b,c.

The bandgap energies (Eg) of these samples (Figure 3c_1_-c_4_) were obtained from the extrapolated intercept on the abscissa (Figure 3b). The estimated bandgap energies of pure P25 TiO_2_, Au/TiO_2_, Pd/TiO_2_ and 1.5Au-0.8Pd/TiO_2_ are 3.54, 3.50, 3.49, and 3.45 eV, respectively. Obviously, the bandgap of the photocatalysts after metal doping is shorter than the pure P25 TiO_2_, wherein the bimetallic Au–Pd/TiO_2_ has the shortest bandgap. This red shift could be attributed to the metal doping and LSPR effect. Although this effect is small, shorter bandgap is beneficial for improving the efficiency of the photocatalyst by harvesting more photons.

**Figure 4:**
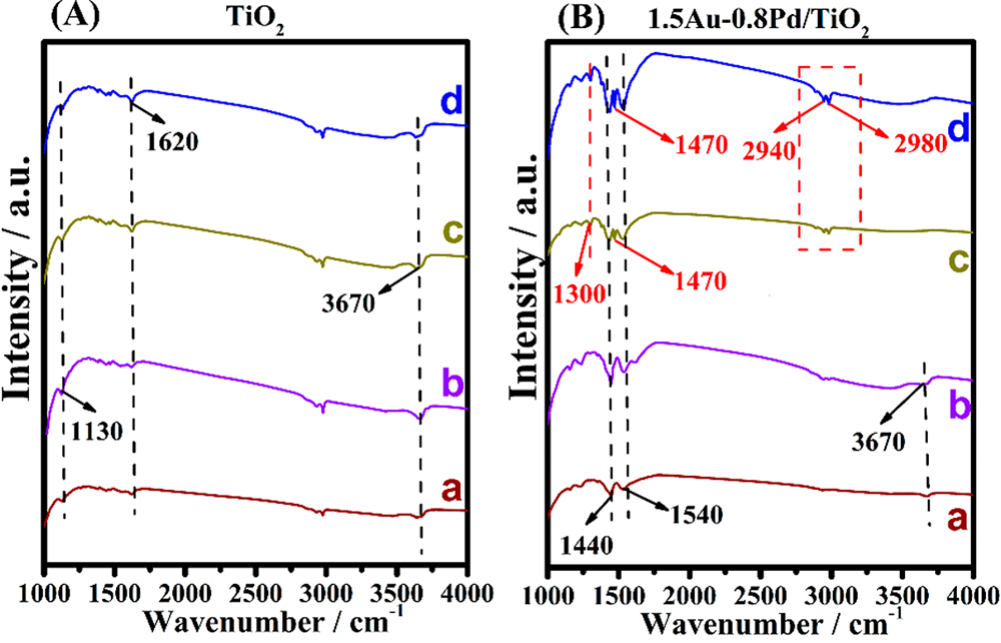
IR spectra of (A) TiO_2_ and (B) 1.5Au-0.8Pd/TiO_2_ with propionic acid adsorbed for (a) shorter and (b) longer time and then irradiated with UV–vis light for (c) 30 and (d) 60 min.

In situ IR spectra were collected to detect the adsorption of carboxylic acids on the photocatalyst surface and the formation of intermediates upon irradiation. The spectra recorded are shown in Figure 4 and Figures S4–S5. The peak at 1130 cm^–1^ is attributed to P25 TiO_2_, while the peak at around 1620 cm^–1^ is attributed to the adsorbed water on the surface, and the one located at 3670 cm^–1^ is ascribed to the adsorbed acid over the TiO_2_ (seen in Figure 4A).^53−55^ The intensity of peaks attributed to propionic acid increases with the prolonged adsorption time, indicating that more propionic acid becomes adsorbed on the surface (Figure 4A (a,b). No differences were found among the IR spectra before and after irradiation for the TiO_2_ sample. As for the 1.5Au-0.8Pd/TiO_2_ catalyst (Figure 4B), the peaks at 1440 and 1540 cm^–1^ are attributed to the symmetric stretching vibration (*v*_s_) and asymmetric stretching vibration (*v*_as_) of COO- bands of propionic acid, respectively.^53,56^ This indicates the formation of a chemical bond between the carboxylic group and the Brønsted acid site (Ti–OH) of the TiO_2_ surface.^57^ Noteworthy, new peaks located at 1300 cm^–1^, 1470 cm^–1^, 2940 and 2980 cm^–1^ are observed, while the peak at 3670 cm^–1^ disappeared upon irradiation. Disappearance of the 3670 cm^–1^ peak agrees with the occurrence of photodecarboxylation. Peaks at 1300, 1470, 2940 and 2980 cm^–1^ are attributed to the bending vibration of −CH_2_–, *v*_s_ and *v*_as_ of alkyl radicals, respectively.^58^ This demonstrates that alkyl radicals were formed on the surface of 1.5Au-0.8Pd/TiO_2_ after UV–vis light irradiation. Meanwhile, it was found that the intensity of these peaks increased along with the irradiation time (Figure 4B (c,d)). Similar results were also obtained over Au/TiO_2_ and Pd/TiO_2_ (Figures S4–S5).

### 3.2 Photocatalytic Activity

Photocatalytic experiments were performed initially in dodecane (10 mL) dissolving hexanoic acid (30 mg) and using 20 mg of photocatalyst, irradiating with the full output of a 300 W Xe lamp through quartz. Prior to the irradiation, the suspension was deaerated with Ar, and the reactor was sealed with an overpressure of 0.5 bar Ar. In normal cases, the formation of pentane was observed. The results after 4 and 11 h irradiation are presented in Table S2 and Table 1. As can be seen in Table 1, those bimetallic samples with Pd loading equal to or higher than 0.8 wt % perform better than the photocatalysts without metal nanoparticles or containing individual Au or Pd nanoparticles. The 1.5Au-0.2Pd/TiO_2_ catalyst with lesser Pd loading performs even worse than Pd/TiO_2_ with individual Pd loading. This can be caused by the much lower loading of Pd (0.2 wt %) in 1.5Au-0.2Pd/TiO_2_ compared to the Pd/TiO_2_ (0.8 wt %). The other bimetallic catalysts with different Au–Pd ratios show similar catalytic performance, wherein the 1.5Au-0.8Pd/TiO_2_ catalyst combines a low Pd content and high photodecarboxylation activity. In fact, 1.5Au-5Pd/TiO_2_ with much higher Pd content and also a core (Au)@shell (Pd) Au–Pd structure exhibits similar photocatalytic performance but at much higher Pd cost. From this initial screening, the samples Au/TiO_2_, Pd/TiO_2_ and 1.5Au-0.8Pd/TiO_2_ were selected for further study because of their performance, while 1.5Au-0.8Pd/TiO_2_ contains the lowest Pd content. For comparison, the performance of 1.5Au0.8Pd alloy/TiO_2_, a 1:1 mechanical mixture of 1.5Au/TiO_2_ and 0.8Pd/TiO_2_ was also examined. The results are listed in Table S2. In all cases, the catalytic performance was much worse than that of 1.5Au(core)-0.8Pd(shell)/TiO_2_, which demonstrates that the synergistic effect endowed by the Au(core)-Pd(shell) structure is essential for the efficient photodecarboxylation of hexanoic acid.

**Table 1:** Photocatalytic Data at 11 h Irradiation of Hexanoic Acid Photodecarboxylation in the Presence of Different Photocatalysts

Catalysts	Conversion (%)^a^	Pentane Yield (%)	Pentane Selectivity (%)	Initial Rate (mol·g_cat_^–1^·h^–1^)^b^
P25 TiO_2_	16.9	15.9	94.1	–
Au/TiO_2_	70.6 ± 2.9	67.9	96.2	0.63
Pd/TiO_2_	90.0 ± 2.0	86.8	96.4	0.77
1.5Au-0.2Pd/TiO_2_	72.7 ± 3.1	69.9	96.3	–
1.5Au-0.8Pd/TiO_2_	96.3 ± 1.6	95.6	99.3	0.86
1.5Au-1.8Pd/TiO_2_	97.5 ± 0.9	96.6	99.1	–
1.5Au-5Pd/TiO_2_	97.5 ± 1.7	96.9	99.3	–

a Data correspond to the average of three independent experiments.

b The initial reaction rates of catalysts were calculated based on the metal amount and activity data at 0.5 h as indicated in eq 4 in the experimental section.

**Figure 5:**
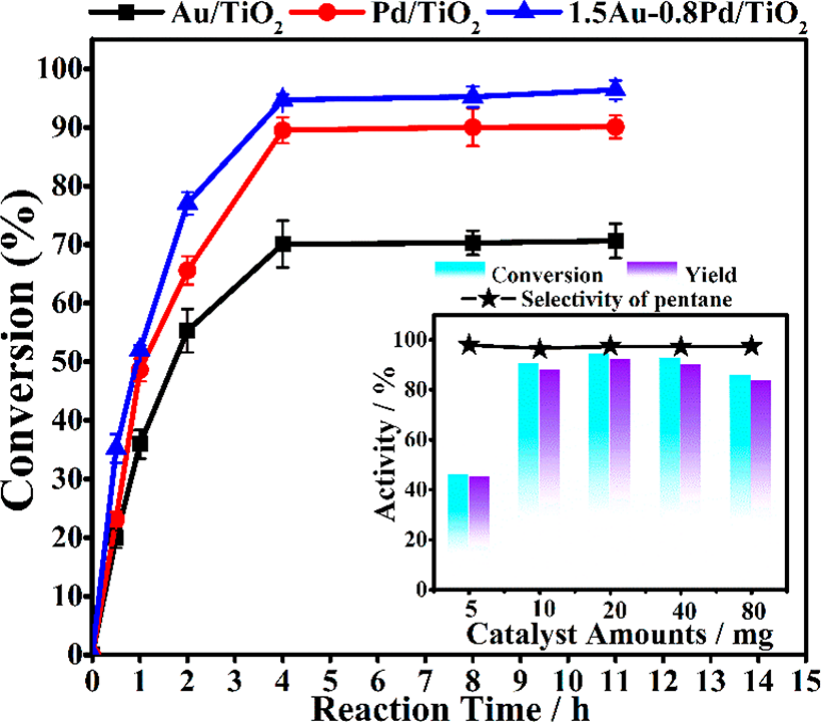
Temporal evolution of hexanoic acid decarboxylation for Au/TiO_2_, Pd/TiO_2_ and 1.5Au-0.8Pd/TiO_2_. (Inset figure is the effect of 1.5Au-0.8Pd/TiO_2_ catalyst amounts on the catalytic results). Reaction conditions: 30 mg of hexanoic acids, 10 mL of dodecane, room temperature, 300 W Xe lamp, 2 h, 20 mg of catalysts, 0.5 bar H_2_.

The temporal evolution of hexanoic acid decarboxylation for Au/TiO_2_, Pd/TiO_2_ and 1.5Au-0.8Pd/TiO_2_ is presented in Figure 5 that further illustrates the benefit of the Au(core)-Pd(shell)/TiO_2_ bimetallic catalyst in comparison to the individual metals. The superior activity of 1.5Au-0.8Pd/TiO_2_ can be explained by a combination of its narrower bandgap energy and the more efficient catalytic activity of the Au(core)-Pd(shell) nanoparticle.

From this temporal profile, irradiation time for 4 h was selected, since using the presented conditions, high conversion values were achieved at this time. The initial reaction rates of different catalysts were calculated based on the metal amounts, and the corresponding values are given in Table 1. It is found that the bimetallic Au(core)-Pd(shell) catalyst shows higher initial reaction rate, indicating its higher intrinsic reactivity toward the photodecarboxylation of hexanoic acid than other catalysts with only mono metal loaded. The influence of photocatalyst weight on hexanoic acid conversion and pentane selectivity was also studied under the same conditions using 5 to 80 mg of 1.5Au-0.8Pd/TiO_2_ in 10 mL dodecane (inset of Figure 5). Photocatalytic results show that conversion increases notably from 5 to 10 mg, becoming then stable even up to a considerable increase in mass excess of 80 mg. From this experiment, an optimal 20 mg photocatalyst mass in 10 mL of dodecane was used for all the experiments. Noteworthy is the fact that decane was not detectable even though the reaction was carried out in the absence of H_2_ as the H atom donor (see Figure S6). Also, an overpressure of Ar was not necessary, and identical conversion and pentane selectivity were observed for irradiations under atmospheric pressure.

The influence of the solvent on hexanoic acid photodecarboxylation was then studied, and the corresponding results are presented in Figure S7. The best result was obtained using dodecane as the solvent, while toluene as the solvent gave the worst result, probably due to an internal filter effect of this aromatic compound in the UV region. Photodecarboxylation seems to be quenched considerably using THF, DMF or dichloroethane (DCE) as solvents, reaching hexanoic acid conversions below 20%, which is probably due to the competition of this solvent with the substrate for trapping of holes (THF) or photogenerated electrons (DMF and DCE).

**Figure 6:**
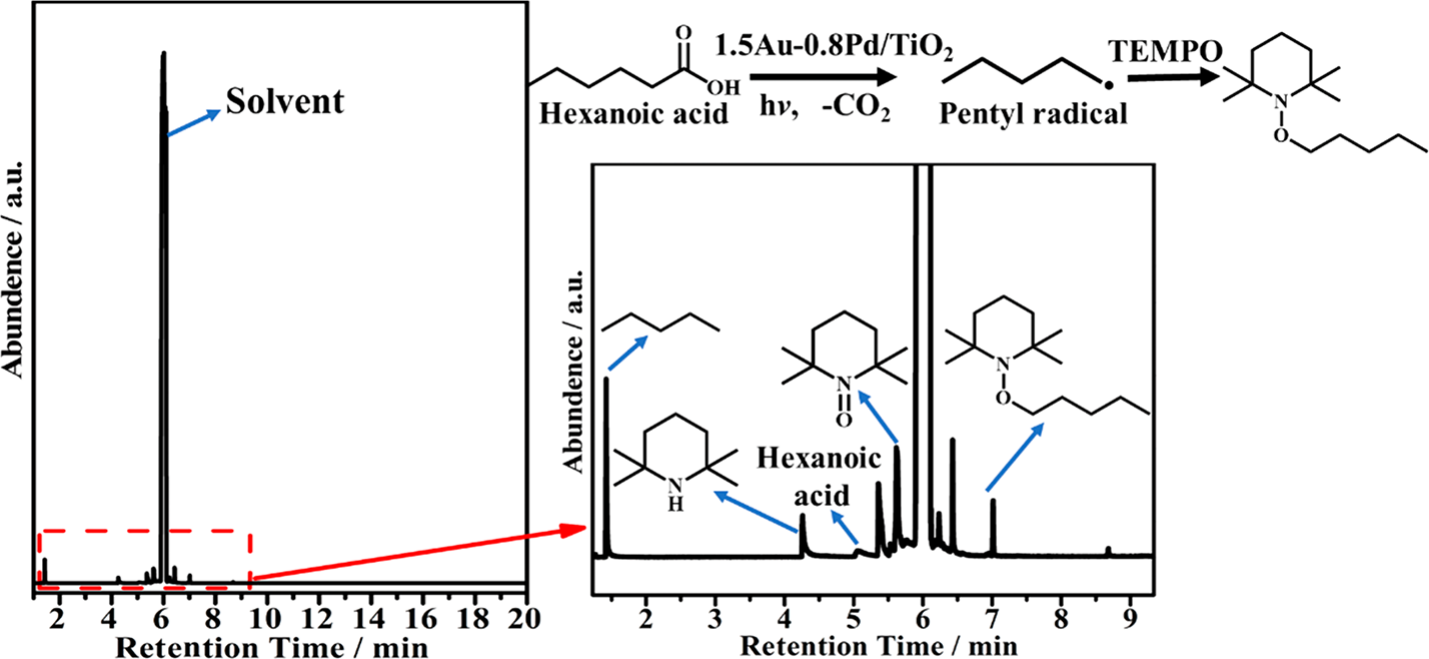
Radical trapping experiment. Products were observed in the photocatalytic decarboxylation of hexanoic acid in the presence of 2,2,6,6-tetramethylpiperidinyloxy (TEMPO) in a H_2_ atmosphere. Reaction conditions: 30 mg hexanoic acids, 10 mL dodecane, room temperature, 300 W Xe lamp, 2 h, 20 mg of catalysts, 0.5 bar H_2_.

To gain some information about the reaction mechanism of the photodecarboxylation, additional experiments were carried out. Thus, no hexanoic acid conversion was observed upon irradiation of hexanoic acid in the absence of photocatalysts or in the presence of 1.5Au-0.8Pd/TiO_2_ but in the dark at 100 °C, meaning that both light and photocatalyst are necessary in the photodecarboxylation process (Table S3). The influence of the wavelength range on the photocatalytic activity was also conducted, as shown also in Table S3. Using cutoff filters for radiations of wavelengths shorter than 360 or 455 nm, both conversions and alkane yield decreased. Moreover, an experiment using TEMPO as a carbon radical trapping agent reveals the formation of pentyl radical as intermediate. This result (Figure 6) is in accordance with the in situ IR measurements that show the presence of alkyl groups during the photodecarboxylation process. These pentyl radicals would arise from the decarboxylation of hexanoate promoted by photogenerated holes.

**Figure 7:**
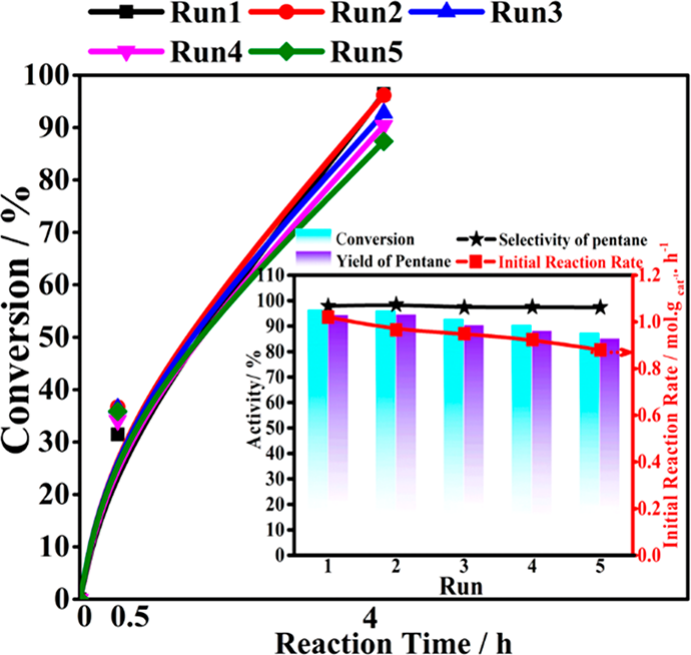
Stability tests for hexanoic acid conversion over 1.5Au-0.8Pd/TiO_2_ photocatalyst. Reaction conditions: 30 mg of hexanoic acids, 10 mL of dodecane, room temperature, 4 h; catalyst was recovered after the last run, 0.5 bar H_2_. The inset plot shows activity, CO_2_ conversion, pentane yield and initial reaction rate.

The stability of the 1.5Au-0.8Pd/TiO_2_ catalyst was also assessed, and the results are shown in Figure 7. In the recycling experiments, the solid catalyst was retrieved after each run from the reaction mixture through filtration. It was subsequently washed with dodecane and employed in the next run. The result reveals a slight, gradual decrease in the conversion of hexanoic acid, while the selectivity of the product remains almost constant for each run, at approximately 97.5%. These reusability data indicate the stability of the bimetallic 1.5Au-0.8Pd/TiO_2_ photocatalyst. Moreover, the initial reaction rates measured at 0.5 h across successive runs show nearly constant values, with only a slight decrease (from 0.86 to 0.72 mol. g _cat_^–1^. h^–1^), further exemplifying the stable nature of this Au (core)-Pd(shell)/TiO_2_ bimetallic catalyst. XRD and XPS results of the five times used 1.5Au-0.8Pd/TiO_2_ indicate almost no changes compared with the fresh one (Figures S8–S9). Particularly, no agglomeration or growth of the metal nanoparticles was found by TEM analysis of the used 1.5Au-0.8Pd/TiO_2_ photocatalyst. Moreover, the core–shell structure of the Au–Pd alloy nanoparticles remains (Figure S10). The stability of the photocatalyst nanostructure is closely related to the stable activity results.

The main components in crude bio-oils include saturated fatty acids such as lauric acid, palmitic acid, stearic acid, unsaturated oleic acid, linoleic acid, and corresponding triglycerides. Therefore, we were interested in determining the scope of this core (Au)-Pd (shell)/TiO_2_ bimetallic photocatalyst to efficiently convert long-chain fatty acids that have to be the feedstocks in realizing the photocatalytic conversion of bio-oils into alkanes. Table 2 demonstrates that 1.5Au-0.8Pd/TiO_2_ exhibits a broad substrate scope, allowing for high conversions and yields of C_*n*–1_ alkanes in the photodecarboxylation of various saturated fatty acids. As for the oleic acid, the somewhat lower C_*n*–1_ alkane yield and selectivity are due to the presence of C=C double bonds in the molecule. In this case, the hole-induced alkyl radicals from photodecarboxylation should tend to attack the unsaturated C=C bonds of the substrate and induce polymerization. Complex raw bio-oils constituted by a mixture of fatty acids also undergo photodecarboxylation. Thus, crude bio-oils such as jatropha oil, wasted cooking oil and wasted hot-pot oil become also converted. Due to the complex composition of crude raw bio-oils, it is difficult to calculate the conversion, yield as well as alkanes selectivity. However, according to the GC-MS spectra of corresponding products in these three cases (Figure S11), various alkanes not present in the feedstock were detected after photocatalytic irradiation, implying the capability of the synthesized core (Au)-shell (Pd)/TiO_2_ bimetallic catalyst to photodecarboxylate fatty acids present in crude bio-oils, converting them into alkanes.

**Table 2:** Photodecarboxylation of Fatty Acids and Bio-oils over 1.5Au-0.8Pd/TiO_2_^a^

Feedstock	Products	Conversion (%)	Yield (%)	Selectivity (%)
Lauric acid	Undecane	100	94.7	94.7
Palmitic acid	Pentadecane	100	97.7	97.7
Stearic acid	Heptadecane	100	94.3	94.3
Oleic acid	Heptadecane	95.5	77.5	81.2

a These reactions are conducted in a H_2_ atmosphere (0.5 bar) under UV–visible light irradiation for 4 h.

### 3.3 Computational Results

To gain some insight into the reaction sites and mechanism of this photodecarboxylation reaction on 1.5Au-0.8Pd/TiO_2_ (denoted for the calculations as Au@Pd), periodic DFT calculations were performed. Here, we focus on the thermodynamic process of the surface reactions, including the dissociation of carboxylic acid and the formation of alkane. The photon absorption event or the kinetic process of the subsequent charge migration is not considered in the calculations, since the carrier dynamics of metals supported on titania and the Schottky barrier between metal titania have been extensively studied elsewhere.^59−62^ Herein, photoexcitation and charge migration are considered to generate electrochemical potentials for catalyzing carboxylic acid decomposition and transformation to alkane. Butyric acid is regarded as a model carboxylic acid for the DFT calculation. Based on the XRD pattern that shows that the main exposed lattice plane in P25 is anatase TiO_2_(101), calculations were made for a model of this 101 anatase plane.

Based on the calculated results, the TiO_2_ (101) facet and metal surfaces (Au@Pd, Au and Pd) show a strong adsorption energy of carboxylic acids (Figure S12). TiO_2_ (101) surface is considered as the oxidation reaction site due to its larger reaction surface area and higher hole concentration. The probable oxidation half-reaction mechanism steps are as follows:
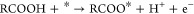
6
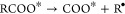
7where RCOOH is the carboxylic acid, the asterisk (*) sign denotes the anatase TiO_2_ (101) surface, RCOO* and COO* denote adsorbed intermediate species, and R^•^ denotes the free alkyl radical. We obtain the energy of H^+^ + e^–^ implicitly by referencing it to the energy of H_2_ using the computational values for the hydrogen electrode.^63^ In this oxidation reaction, the first step is the adsorption of carboxylic acid. The adsorption energy is calculated to be −0.71 eV (Figure S13), which indicates that the carboxylic acid molecule strongly interacts with the TiO_2_ surface. This interaction was also demonstrated by calculating Hirshfeld charge distribution, and it was found that the carboxylic acid molecule presents a more positive charge (0.26 |e|) when it adsorbs on the TiO_2_ surface. Projected density of states (PDOS) shows the Ti 3d and O 2p orbitals dominate the electrons density under the Fermi level (Figure S13 a), and the O 2p orbital of *RCOOH intermediate shows a high degree of overlap with TiO_2_ p and d-orbitals between 0 and −4 eV under the Fermi level (Figure S13 b). This overlap suggests a significant interaction between the O 2p orbital in RCOOH and Ti 3d and the O 2p orbitals in TiO_2_. All the above results reveal the strong interaction between carboxylic acid and the TiO_2_ surface, which benefits the subsequent reaction.

**Figure 8:**
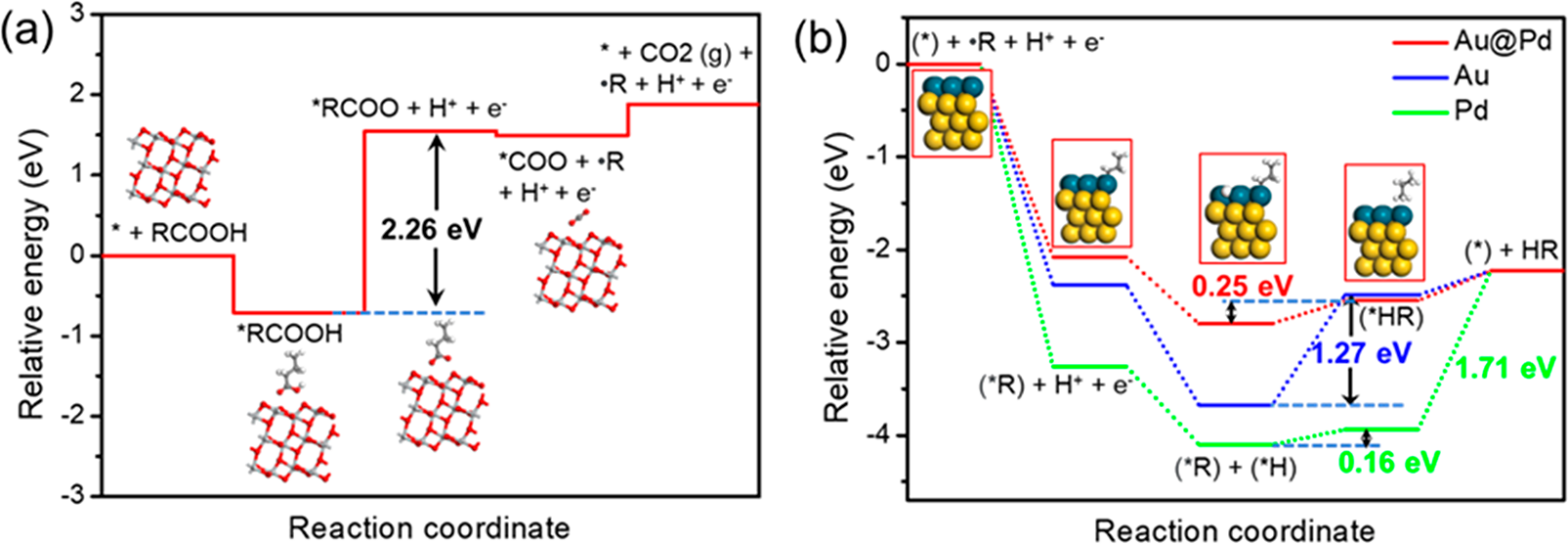
Potential energy diagram for the (a) oxidation half-reaction on the TiO_2_ (101) surface and (b) reduction half-reaction on Au@Pd, Au and Pd.

The next step is dissociative dehydrogenation of the carboxylic acid. Carboxylic acid is thought to be deprotonated by hole oxidation.^64^ In this work, we have considered overpotential as the important activity descriptor since it is of considerable interest from an experimental perspective. The overpotential obtained from the energy difference has been used as activity descriptor, because it follows the same trend to kinetic barriers owing to the Brønsted–Evans–Polanyi (BEP) relation between free energy and activation barriers.^65,66^ Here, we use reaction enthalpies instead of free energies to compute the reaction mechanism since frequency calculations for this model are computationally very expensive. In addition, for long-chain carboxylic acids and alkanes, the vibrational frequency is relatively less affected by the adsorption and reaction. Hence, we believe that our energy-profile-based analysis is reasonable to explain the activity of this system. As shown in Figure 8a, the calculated overpotential for RCOOH deprotonation is 2.26 eV (eq 6), subsequent C–C bond breaking to generate free radical R^•^ is exergonic (eq 7), and the TiO_2_ is recovered by desorption of CO_2_ with moderate energy (0.39 eV). Therefore, photogenerated holes in the valence band (3.08 eV)^67^ provide enough driving force to compensate the RCOOH deprotonation overpotential.

On the other hand, the probable reduction half-reaction mechanism steps have also been considered:
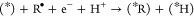
8
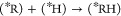
9As shown in Figure S14, the adsorption and reduction of H^+^ on TiO_2_ (101) surface is endergonic (*E*_ads_ = 0.61 eV), while this reduction process of the proton on Au@Pd (*E*_ads_= −0.95 eV), Au (*E*_ads_= −0.65 eV) or Pd (*E*_ads_= −1.58 eV) is thermodynamically favorable. Furthermore, it is commonly known that the metal has a higher work function; thus, the photogenerated electrons formed on the conduction band of TiO_2_ tend to migrate to the metal nanoparticle. In addition, the adsorption of ^•^R on Au@Pd (*E*_ads_= −2.08 eV), Au (*E*_ads_= −2.38 eV) or Pd (*E*_ads_= −3.26 eV) is also more thermodynamically favorable than that on the TiO_2_ (101) surface (*E*_ads_= −0.68 eV) (Figure S15). Therefore, the metal nanoparticle is considered as the reduction reaction site. The reduction reactions to form HR on Au@Pd, Pd and Au are all considered. As shown in Figure 8b, Pd and Au exhibit much stronger adsorption of H^•^ and R^•^, and the subsequent overpotential for H–R bond formation indicates that Au@Pd (0.25 eV) and Pd (0.16 eV) have a lower overpotential compared to only Au (1.27 eV). However, it is worth noting that Pd exhibits a higher H-R desorption energy (1.71 eV), which results in a higher initial reaction rate and conversion than that of Au, but still lower than that of Au@Pd. These findings confirm that Au@Pd should possess superior activity for alkane formation compared to Pd and Au.

**Scheme 2:**
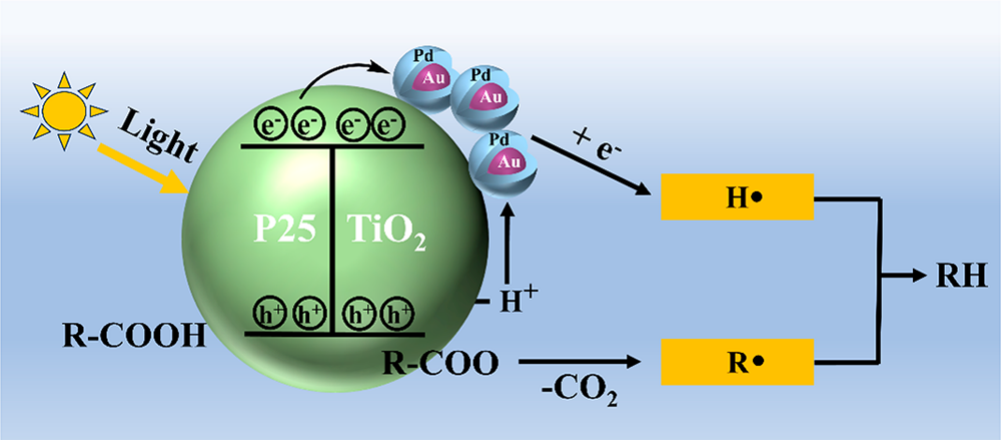
Diagram of the Proposed Reaction Mechanism for Photodecarboxylation of Carboxylic Acid over 1.5Au-0.8Pd/TiO_2_ Catalyst

Combining the experimental and DFT calculation results, we propose a plausible mechanism for the photodecarboxylation of carboxylic acid over 1.5Au-0.8Pd/TiO_2_ in Scheme 2.

First, light irradiation over P25 TiO_2_ leads to charge separation with the generation of electrons and holes. Carboxylic acid molecules become dissociated, and the resulting carboxylate strongly adsorbed on the TiO_2_ surface. The photogenerated holes on the TiO_2_ surface will be quenched by the carboxylate groups in a kind of ligand to metal electron transfer to form RCOO^•^ intermediates. RCOO^•^ intermediates adsorbed strongly on the TiO_2_ surface undergo decarboxylation to form CO_2_ and alkyl radicals. H^+^ generated in the carboxylic acid dissociation would migrate to Au–Pd nanoparticles, combining with photoinduced electrons to form H^•^. Finally, the alkyl radical bonds with H^•^ to form C_*n*–1_ alkanes. Au–Pd nanoparticles would play several roles including: (i) the increase in the charge separation efficiency by accepting electrons from TiO_2_, (ii) the generation of highly mobile H^•^ atoms through the photocatalyst surface by reducing H^+^ protons, and (iii) promote the evolution of pentane by coupling penntyl radical and H^•^ atoms. The fact that the *n*-pentane selectivity does not decrease in the photocatalytic reactions in the absence of H_2_ as an additional source of hydrogen indicates that the protons derived from hexanoic acid dissociation can be efficiently reduced to H^•^ atoms.

## 4 Conclusions

In summary, the synthesized Au (core)-Pd (shell)/TiO_2_ catalyst was proven to be a novel UV–vis light responsive photocatalyst, demonstrating excellent activity and stability for the photodecarboxylation of hexanoic acid (conversion for 94.7% and near 100% pentane selectivity) as well as other fatty acids and even raw crude bio-oils. The unique structure characteristics endow the Au–Pd bimetallic TiO_2_ photocatalyst with distinctive electronic effects and high charge-separation efficiency, thus exhibiting superior photocatalytic activity compared with pure TiO_2_, or monometallic Au and Pd catalysts. DFT calculations revealed that oxidative decarboxylation reactions of carboxylic acids and hydrogen bonding of alkyl radicals are likely to occur on TiO_2_ and metal nanoparticles, respectively. Meanwhile, it revealed that the adsorption for H is weaker and the overpotential of HR formation is lower on Au(core)-Pd(shell)/TiO_2_ catalysts, which is favorable for forming C_*n*–1_ alkanes. The present study shows a promising way to upgrade the low-value biomass-derived carboxylic acids to high-value alkanes.

## Supporting Information Available

High-resolution TEM images, EDX-Mapping of prepared catalysts; IR spectra with propionic acid adsorbed on Au/TiO_2_ and Pt/TiO_2_; effect of atmosphere, solvent on the photoactivity; XRD, XPS patterns and TEM images as well as EDX-Mapping of used 1.5Au-0.8Pd/TiO_2_ catalysts; GC-MS spectra of crude oils and corresponding products by photocatalytic decarboxylation; optimized geometry models and corresponding adsorption energies of RCOOH adsorbed on different surfaces; PDOS of Ti and O orbitals in TiO_2_ adsorbed sites and PDOS of O, H, and C orbitals in RCOOH and TiO_2_ orbital in TiO_2_ adsorbed sites; Optimized structural models and corresponding adsorption energies of H and R^•^ on different surfaces; ICP-AES data; other activity performance (PDF)

## References

[ref1] Taufiqurrahmi N.; Bhatia S. Catalytic cracking of edible and non-edible oils for the production of biofuels. Energy Environ. Sci. 2011, 4 (4), 1087–1112. 10.1039/c0ee00460j.

[ref2] Wang M.; Dewil R.; Maniatis K.; Wheeldon J.; Tan T.; Baeyens J.; Fang Y. Biomass-derived aviation fuels: Challenges and perspective. Prog. Energy Combust. Sci. 2019, 74, 31–49. 10.1016/j.pecs.2019.04.004.

[ref3] Žula M.; Grilc M.; Likozar B. Hydrocracking, hydrogenation and hydro-deoxygenation of fatty acids, esters and glycerides: Mechanisms, kinetics and transport phenomena. Chemical Engineering Journal 2022, 444, 136564–136588. 10.1016/j.cej.2022.136564.

[ref4] Qu L.; Jiang X.; Zhang Z.; Zhang X.-g.; Song G.-y.; Wang H.-l.; Yuan Y.-p.; Chang Y.-l. A review of hydrodeoxygenation of bio-oil: model compounds, catalysts, and equipment. Green Chem. 2021, 23 (23), 9348–9376. 10.1039/D1GC03183J.

[ref5] Pritchard J.; Filonenko G. A.; van Putten R.; Hensen E. J.; Pidko E. A. Heterogeneous and homogeneous catalysis for the hydrogenation of carboxylic acid derivatives: history, advances and future directions. Chem. Soc. Rev. 2015, 44 (11), 3808–33. 10.1039/C5CS00038F. 25941799

[ref6] Wang M.; Zhou H.; Wang F. Photocatalytic Production of Syngas from Biomass. Acc. Chem. Res. 2023, 56 (9), 1057–1069. 10.1021/acs.accounts.3c00039. 37043679

[ref7] Huang Z.; Zhao Z.; Zhang C.; Lu J.; Liu H.; Luo N.; Zhang J.; Wang F. Enhanced photocatalytic alkane production from fatty acid decarboxylation via inhibition of radical oligomerization. Nature Catalysis 2020, 3 (2), 170–178. 10.1038/s41929-020-0423-3.

[ref8] Shi J.; Yuan T.; Zheng M.; Wang X. Metal-Free Heterogeneous Semiconductor for Visible-Light Photocatalytic Decarboxylation of Carboxylic Acids. ACS Catal. 2021, 11 (5), 3040–3047. 10.1021/acscatal.0c05211.

[ref9] Long F.; Cao X.; Liu P.; Jiang X.; Jiang J.; Zhang X.; Xu J. Tuning charge transfer of Pt cluster by support defects in Pt/TiO2 for photocatalytic conversion of fatty acid into diesel-like alkane. Journal of Cleaner Production 2022, 375, 133975–133985. 10.1016/j.jclepro.2022.133975.

[ref10] Heciak A.; Morawski A. W.; Grzmil B.; Mozia S. Cu-modified TiO_2_ photocatalysts for decomposition of acetic acid with simultaneous formation of C_1_-C_3_ hydrocarbons and hydrogen. Applied Catalysis B: Environmental 2013, 140–141, 108–114. 10.1016/j.apcatb.2013.03.044.

[ref11] Schwarz J.; König B. Decarboxylative reactions with and without light - a comparison. Green Chem. 2018, 20 (2), 323–361. 10.1039/C7GC02949G.

[ref12] Du X.; Liu J.; Li D.; Xin H.; Lei X.; Zhang R.; Zhou L.; Yang H.; Zeng Y.; Zhang H.; Zheng W.; Wen X.; Hu C. Structural and electronic effects boosting Ni-doped Mo_2_C catalyst toward high-efficiency C-O/C-C bonds cleavage. J. Energy Chem. 2022, 75, 109–116. 10.1016/j.jechem.2022.08.024.

[ref13] Yang H.; Zeng Y.; Zhou Y.; Du X.; Li D.; Hu C. One-step synthesis of highly active and stable Ni-ZrO_2_ catalysts for the conversion of methyl laurate to alkanes. J. Catal. 2022, 413, 297–310. 10.1016/j.jcat.2022.06.035.

[ref14] Zhou Y.; Remón J.; Jiang Z.; Matharu A. S.; Hu C. Tuning the selectivity of natural oils and fatty acids/esters deoxygenation to biofuels and fatty alcohols: A review. Green Energy Environ. 2023, 8, 722–743. 10.1016/j.gee.2022.03.001.

[ref15] Du X.; Peng Y.; Albero J.; Li D.; Hu C.; Garcia H. Synthetic Fuels from Biomass: Photocatalytic Hydrodecarboxylation of Octanoic Acid by Ni Nanoparticles Deposited on TiO_2_. ChemSusChem 2022, 15 (2), e202102107 10.1002/cssc.202102107. 34841693

[ref16] Chen X.; Shen S.; Guo L.; Mao S. S. Semiconductor-based Photocatalytic Hydrogen Generation. Chem. Rev. 2010, 110, 6503–6570. 10.1021/cr1001645. 21062099

[ref17] Wang Y.; Silveri F.; Bayazit M. K.; Ruan Q.; Li Y.; Xie J.; Catlow C. R. A.; Tang J. Bandgap Engineering of Organic Semiconductors for Highly Efficient Photocatalytic Water Splitting. Adv. Energy Mater. 2018, 8 (24), 1801084–1801093. 10.1002/aenm.201801084.

[ref18] Wu X.; Li J.; Xie S.; Duan P.; Zhang H.; Feng J.; Zhang Q.; Cheng J.; Wang Y. Selectivity Control in Photocatalytic Valorization of Biomass-Derived Platform Compounds by Surface Engineering of Titanium Oxide. Chem. 2020, 6 (11), 3038–3053. 10.1016/j.chempr.2020.08.014.

[ref19] Cao S.; Chan T.-S.; Lu Y.-R.; Shi X.; Fu B.; Wu Z.; Li H.; Liu K.; Alzuabi S.; Cheng P.; Liu M.; Li T.; Chen X.; Piao L. Photocatalytic pure water splitting with high efficiency and value by Pt/ porous brookite TiO_2_ nanoflutes. Nano Energy 2020, 67, 104287 10.1016/j.nanoen.2019.104287.

[ref20] Wang L.; Xiao Z.; Liu Y.; Cao S.; Ma Z.; Piao L. Mesoporous TiO_2_ mixed crystals for photocatalytic pure water splitting. Science China Materials 2020, 63 (5), 758–768. 10.1007/s40843-019-1253-8.

[ref21] Ao C. H.; Lee S. C. Indoor air purification by photocatalyst TiO_2_ immobilized on an activated carbon filter installed in an air cleaner. Chem. Eng. Sci. 2005, 60 (1), 103–109. 10.1016/j.ces.2004.01.073.

[ref22] Cybula A.; Priebe J. B.; Pohl M.-M.; Sobczak J. W.; Schneider M.; Zielińska-Jurek A.; Brückner A.; Zaleska A. The effect of calcination temperature on structure and photocatalytic properties of Au/Pd nanoparticles supported on TiO_2_. Applied Catalysis B: Environmental 2014, 152–153, 202–211. 10.1016/j.apcatb.2014.01.042.

[ref23] Bowker M.; O’Rourke C.; Mills A. The Role of Metal Nanoparticles in Promoting Photocatalysis by TiO_2_. Top Curr. Chem. (Cham) 2022, 380 (3), 17 10.1007/s41061-022-00373-x. 35237896 PMC8891105

[ref24] Meng A.; Zhang L.; Cheng B.; Yu J. Dual Cocatalysts in TiO_2_ Photocatalysis. Adv. Mater. 2019, 31 (30), e1807660 10.1002/adma.201807660. 31148244

[ref25] Caudillo-Flores U.; Muñoz-Batista M. J.; Fernández-García M.; Kubacka A. Bimetallic Pt-Pd co-catalyst Nb-doped TiO_2_ materials for H_2_ photo-production under UV and Visible light illumination. Applied Catalysis B: Environmental 2018, 238, 533–545. 10.1016/j.apcatb.2018.07.047.

[ref26] Zielińska-Jurek A. Progress, Challenge, and Perspective of Bimetallic TiO_2_-Based Photocatalysts. J. Nanomater. 2014, 2014, 1–17. 10.1155/2014/208920.

[ref27] Haldar K. K.; Kundu S.; Patra A. Core-size-dependent catalytic properties of bimetallic Au/Ag core-shell nanoparticles. ACS Appl. Mater. Interfaces 2014, 6 (24), 21946–53. 10.1021/am507391d. 25456348

[ref28] Wang H.; Wang C.; Yan H.; Yi H.; Lu J. Precisely-controlled synthesis of Au@Pd core-shell bimetallic catalyst via atomic layer deposition for selective oxidation of benzyl alcohol. J. Catal. 2015, 324, 59–68. 10.1016/j.jcat.2015.01.019.

[ref29] Shi D.; Sadier A.; Girardon J.-S.; Mamede A.-S.; Ciotonea C.; Marinova M.; Stievano L.; Sougrati M. T.; La Fontaine C.; Paul S.; Wojcieszak R.; Marceau E. Probing the core and surface composition of nanoalloy to rationalize its selectivity: Study of Ni-Fe/SiO_2_ catalysts for liquid-phase hydrogenation. Chem. Catalysis 2022, 2 (7), 1686–1708. 10.1016/j.checat.2022.04.009.

[ref30] Fan Y.; Girard A.; Waals M.; Salzemann C.; Courty A. Ag@Pt Core-Shell Nanoparticles for Plasmonic Catalysis. ACS Applied Nano Materials 2023, 6 (2), 1193–1202. 10.1021/acsanm.2c04767.

[ref31] Zong Z.; Xu K.; Li D.; Tang Z.; He W.; Liu Z.; Wang X.; Tian Y. Peptide templated Au@Pd core-shell structures as efficient bi-functional electrocatalysts for both oxygen reduction and hydrogen evolution reactions. J. Catal. 2018, 361, 168–176. 10.1016/j.jcat.2018.02.020.

[ref32] Grirrane A.; Corma A.; Garcia H. Gold-Catalyzed Synthesis of Aromatic Azo Compounds from Anilines and Nitroaromatics. Science 2008, 322, 1661–1664. 10.1126/science.1166401. 19074342

[ref33] Segall M D; Lindan P. J D; Probert M J; Pickard C J; Hasnip P J; Clark S J; Payne M C MCPayne, First-principles simulation: ideas, illustrations and the CASTEP code. J. Phys.: Condens. Matter 2002, 14, 2717–2744. 10.1088/0953-8984/14/11/301.

[ref34] Perdew J. P.; Burke K.; Ernzerhof M. Generalized Gradient Approximation Made Simple. Phys. Rev. Lett. 1996, 77, 3865–3868. 10.1103/PhysRevLett.77.3865. 10062328

[ref35] Tkatchenko A.; Scheffler M. Accurate molecular van der Waals interactions from ground-state electron density and free-atom reference data. Phys. Rev. Lett. 2009, 102 (7), 073005 10.1103/PhysRevLett.102.073005. 19257665

[ref36] Yu H.; Yang X.; Wu Y.; Guo Y.; Li S.; Lin W.; Li X.; Zheng J. Bimetallic Ru-Ni/TiO_2_ catalysts for hydrogenation of N-ethylcarbazole: Role of TiO_2_ crystal structure. Journal of Energy Chemistry 2020, 40, 188–195. 10.1016/j.jechem.2019.04.009.

[ref37] Shi D.; Liu J.; Sun R.; Ji S.; Rogers S. M.; Connolly B. M.; Dimitratos N.; Wheatley A. E. H. Preparation of bifunctional Au-Pd/TiO_2_ catalysts and research on methanol liquid phase one-step oxidation to methyl formate. Catal. Today 2018, 316, 206–213. 10.1016/j.cattod.2018.01.033.

[ref38] Kruse N.; Chenakin S. XPS characterization of Au/TiO_2_ catalysts: Binding energy assessment and irradiation effects. Applied Catalysis A: General 2011, 391 (1–2), 367–376. 10.1016/j.apcata.2010.05.039.

[ref39] Sun L.; Ren X.; Yin Z.; Jing H.; Zhang M.; Zhou X.; Sun X.; Su H.; Qi C. Promoting effect of trace amount of Pd for p-CNB hydrogenation in Pd-Au/TiO_2_ bimetallic catalyst. Appl. Surf. Sci. 2023, 623, 157107–157114. 10.1016/j.apsusc.2023.157107.

[ref40] Wu P.; Cao Y.; Zhao L.; Wang Y.; He Z.; Xing W.; Bai P.; Mintova S.; Yan Z. Formation of PdO on Au-Pd bimetallic catalysts and the effect on benzyl alcohol oxidation. J. Catal. 2019, 375, 32–43. 10.1016/j.jcat.2019.05.003.

[ref41] Tabakova T.; Ilieva L.; Petrova P.; Venezia A. M.; Avdeev G.; Zanella R.; Karakirova Y. Complete benzene oxidation over mono and bimetallic Au-Pd catalysts supported on Fe-modified ceria. Chemical Engineering Journal 2015, 260, 133–141. 10.1016/j.cej.2014.08.099.

[ref42] Khder A. S.; Altass H. M.; Jassas R. S.; Al-Rooqi M. M.; Khder M. A.; Morad M.; Gebreil A.; Moussa Z.; Ahmed S. A. Room-Temperature CO Oxidation over Au-Pd Monometallic and Bimetallic Nanoparticle-Supported MgO. ACS Applied Nano Materials 2023, 6 (6), 4243–4252. 10.1021/acsanm.2c05326.

[ref43] Wang P.; Zhan S.; Xia Y.; Ma S.; Zhou Q.; Li Y. The fundamental role and mechanism of reduced graphene oxide in rGO/Pt-TiO_2_ nanocomposite for high-performance photocatalytic water splitting. Applied Catalysis B: Environmental 2017, 207, 335–346. 10.1016/j.apcatb.2017.02.031.

[ref44] Devaraji P.; Gopinath C. S. Pt - g-C_3_N_4_ - (Au/TiO_2_): Electronically integrated nanocomposite for solar hydrogen generation. Int. J. Hydrogen Energy 2018, 43 (2), 601–613. 10.1016/j.ijhydene.2017.11.057.

[ref45] Tudu B.; Nalajala N.; Prabhakar Reddy K.; Saikia P.; Gopinath C. S. Electronic Integration and Thin Film Aspects of Au-Pd/rGO/TiO_2_ for Improved Solar Hydrogen Generation. ACS Appl. Mater. Interfaces 2019, 11 (36), 32869–32878. 10.1021/acsami.9b07070. 31414793

[ref46] Xing M.; Zhang J.; Chen F.; Tian B. An economic method to prepare vacuum activated photocatalysts with high photo-activities and photosensitivities. Chem. Commun. (Camb) 2011, 47 (17), 4947–9. 10.1039/c1cc10537j. 21423955

[ref47] Li G.; Liu C.; Liu Y. Different effects of cerium ions doping on properties of anatase and rutile TiO_2_. Appl. Surf. Sci. 2006, 253 (5), 2481–2486. 10.1016/j.apsusc.2006.05.002.

[ref48] Wei H.; Liu W.; Chen X.; Yang Q.; Li J.; Chen H. Renewable bio-jet fuel production for aviation: A review. Fuel 2019, 254, 115599–115614. 10.1016/j.fuel.2019.06.007.

[ref49] Wu L.; Li F.; Xu Y.; Zhang J. W.; Zhang D.; Li G.; Li H. Plasmon-induced photoelectrocatalytic activity of Au nanoparticles enhanced TiO_2_ nanotube arrays electrodes for environmental remediation. Applied Catalysis B: Environmental 2015, 164, 217–224. 10.1016/j.apcatb.2014.09.029.

[ref50] Zhang S.; Zhao W.; Ren A.; Guo B.; Dong Y.; Deng X.-Q. Insight into surface properties of O_2_ plasma activated Au/TiO_2_ prepared by DPU in CO oxidation. Catal. Today 2019, 337, 110–116. 10.1016/j.cattod.2019.04.020.

[ref51] Liu Q.; Zhang J.; Xing F.; Cheng C.; Wu Y.; Huang C. Plasmon-enhanced and controllable synthesis of azobenzene and hydrazobenzene using Au/TiO_2_ composite. Appl. Surf. Sci. 2020, 500, 144214–144219. 10.1016/j.apsusc.2019.144214.

[ref52] Yu H.; Wang X.; Sun H.; Huo M. Photocatalytic degradation of malathion in aqueous solution using an Au-Pd-TiO_2_ nanotube film. J. Hazard. Mater. 2010, 184 (1–3), 753–758. 10.1016/j.jhazmat.2010.08.103. 20855153

[ref53] Nakayama N.; Hayashi T. Preparation of TiO_2_ nanoparticles surface-modified by both carboxylic acid and amine: Dispersibility and stabilization in organic solvents. Colloids Surf., A 2008, 317 (1–3), 543–550. 10.1016/j.colsurfa.2007.11.036.

[ref54] Li J.; Liu T.; Sui G.; Zhen D. Photocatalytic Performance of a Nd-SiO_2_-TiO_2_ Nanocomposite for Degradation of Rhodamine B Dye Wastewater. J. Nanosci. Nanotechnol. 2015, 15 (2), 1408–15. 10.1166/jnn.2015.9611. 26353664

[ref55] CAPECCHI G.; FAGA M. G.; MARTRA G.; COLUCCIA S.; IOZZI M. F.; COSSI M. Adsorption of CH_3_COOH on TiO_2_:IR and theoretical investigations. Res. Chem. Intermed. 2007, 33, 269–284. 10.1163/156856707779238748.

[ref56] McEntee M.; Tang W.; Neurock M.; Yates J. T. Mechanistic Insights into the Catalytic Oxidation of Carboxylic Acids on Au/TiO_2_: Partial Oxidation of Propionic and Butyric Acid to Gold Ketenylidene through Unsaturated Acids. ACS Catal. 2015, 5 (2), 744–753. 10.1021/cs5014255.

[ref57] Ojamae L.; Aulin C.; Pedersen H.; Kall P.-O. IR and quantum-chemical studies of carboxylic acid and glycine adsorption on rutile TiO_2_ nanoparticles. J. Colloid Interface Sci. 2006, 296 (1), 71–78. 10.1016/j.jcis.2005.08.037. 16165144

[ref58] Wang Y.; Woll C. IR spectroscopic investigations of chemical and photochemical reactions on metal oxides: bridging the materials gap. Chem. Soc. Rev. 2017, 46 (7), 1875–1932. 10.1039/C6CS00914J. 28221385

[ref59] Qian R.; Zong H.; Schneider J.; Zhou G.; Zhao T.; Li Y.; Yang J.; Bahnemann D. W.; Pan J. H. Charge carrier trapping, recombination and transfer during TiO_2_ photocatalysis: An overview. Catal. Today 2019, 335, 78–90. 10.1016/j.cattod.2018.10.053.

[ref60] Maity P.; Mohammed O. F.; Katsiev K.; Idriss H. Study of the Bulk Charge Carrier Dynamics in Anatase and Rutile TiO_2_ Single Crystals by Femtosecond Time-Resolved Spectroscopy. J. Phys. Chem. C 2018, 122 (16), 8925–8932. 10.1021/acs.jpcc.8b00256.

[ref61] Kumaravel V.; Mathew S.; Bartlett J.; Pillai S. C. Photocatalytic hydrogen production using metal doped TiO_2_: A review of recent advances. Applied Catalysis B: Environmental 2019, 244, 1021–1064. 10.1016/j.apcatb.2018.11.080.

[ref62] Wang A.; Wu S.; Dong J.; Wang R.; Wang J.; Zhang J.; Zhong S.; Bai S. Interfacial facet engineering on the Schottky barrier between plasmonic Au and TiO_2_ in boosting the photocatalytic CO_2_ reduction under ultraviolet and visible light irradiation. Chemical Engineering Journal 2021, 404, 127145–127152. 10.1016/j.cej.2020.127145.

[ref63] Nørskov J. K.; Rossmeisl J.; Logadottir A.; Lindqvist L.; Kitchin J. R.; Bligaard T.; Jonsson H. Origin of the Overpotential for Oxygen Reduction at a Fuel-Cell Cathode. J. Phys. Chem. B 2004, 108, 17886–17892. 10.1021/jp047349j. 39682080

[ref64] Zhang H.; Zhou P.; Ji H.; Ma W.; Chen C.; Zhao J. Enhancement of photocatalytic decarboxylation on TiO_2_ by water-induced change in adsorption-mode. Applied Catalysis B: Environmental 2018, 224, 376–382. 10.1016/j.apcatb.2017.10.020.

[ref65] van Santen R. A.; Neurock M.; Shetty S. G. Reactivity Theory of Transition-Metal Surfaces: A Brønsted-Evans-Polanyi Linear Activation Energy-Free-Energy Analysis. Chem. Rev. 2010, 110, 2005–2048. 10.1021/cr9001808. 20041655

[ref66] Sutton J. E.; Vlachos D. G. A Theoretical and Computational Analysis of Linear Free Energy Relations for the Estimation of Activation Energies. ACS Catal. 2012, 2 (8), 1624–1634. 10.1021/cs3003269.

[ref67] Kumar A.; Choudhary P.; Kumar A.; Camargo P. H. C.; Krishnan V. Recent Advances in Plasmonic Photocatalysis Based on TiO_2_ and Noble Metal Nanoparticles for Energy Conversion, Environmental Remediation, and Organic Synthesis. Small 2022, 18 (1), e2101638 10.1002/smll.202101638. 34396695

