# Supplementary material for: Enhanced Fatty Acid Photodecarboxylation over Bimetallic Au–Pd Core–Shell Nanoparticles Deposited on TiO2
Source: ACS Catal. 2023 Nov 8;13(22):15143–54. doi: 10.1021/acscatal.3c03793 (PMC10859932; doi:10.1021/acscatal.3c03793)
Supplement: Supplementary file 1 — cs3c03793_si_001.pdf [file cs3c03793_si_001.pdf]

Supporting Information

Enhanced fatty acid photodecarboxylation over bimetallic Au-Pd core-shell nanoparticles deposited on TiO_2

Huiru Yang,[a,b] *Liang Tian,*[b] *Abdessamad Grirrane,*[b] *Alberto García-Baldoví,*[b] *Jiajun Hu,*[b] *German Sastre,*[b] *Changwei Hu,*[a*] *Hermenegildo García*[b *]

[a] Key Laboratory of Green Chemistry and Technology, Ministry of Education, College of Chemistry, Sichuan University, Chengdu, Sichuan 610064, P. R. China;

[b] Instituto Universitario de Tecnología Química, Consejo Superior de Investigaciones Científicas, Universitat Politecnica de Valencia, 46022 Valencia, Spain;

[*]Email: Hermenegildo García: hgarcia@qim.upv.es, Changwei Hu: changweihu@scu.edu.cn

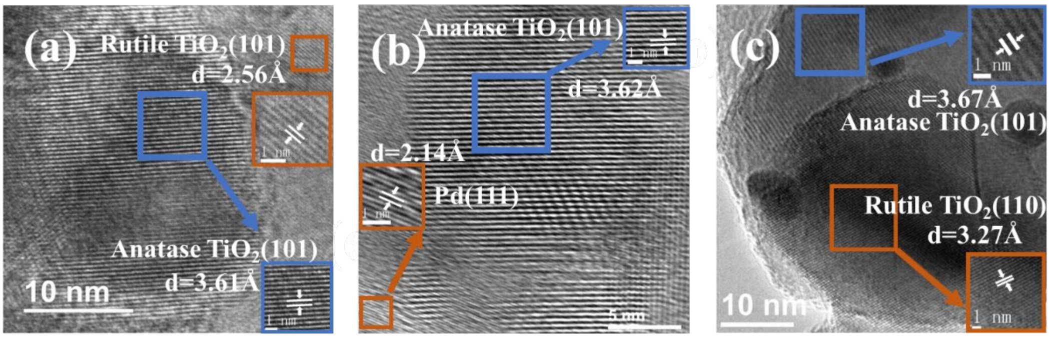

Figure S1. High-resolution TEM images of (a) Au/TiO_2, (b) Pd/TiO_2 and (c) 1.5Au-0.8Pd/TiO_2 catalysts.

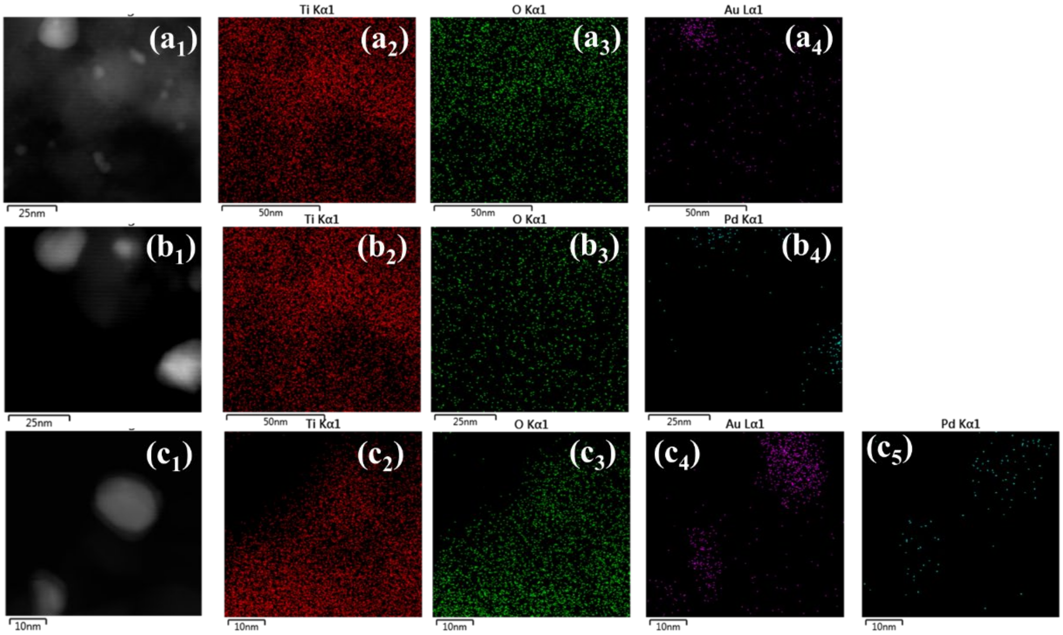

Figure S2. EDX elemental mapping of (a) Au/TiO_2, (b) Pd/TiO_2 and (c)1.5Au-0.8Pd/TiO_2.

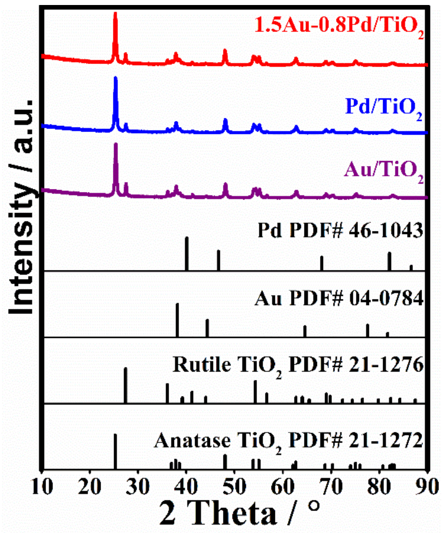

Figure S3. XRD patterns of different samples.

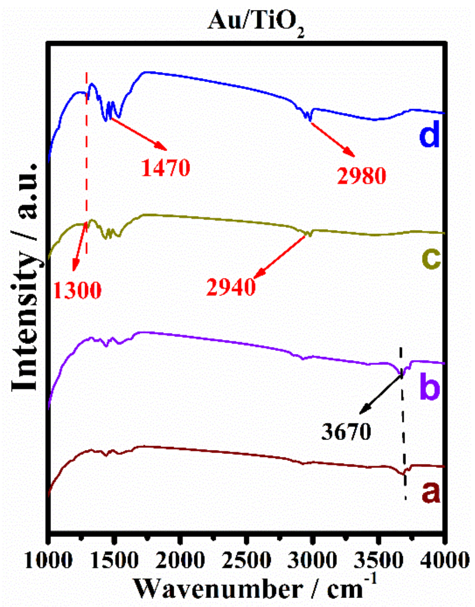

Figure S4. IR spectra of Au/TiO_2 with propionic acid adsorbed for (a) shorter time and (b) longer time, and then irradiated with UV-Vis light for (c) 30 min and (d) 60 min.

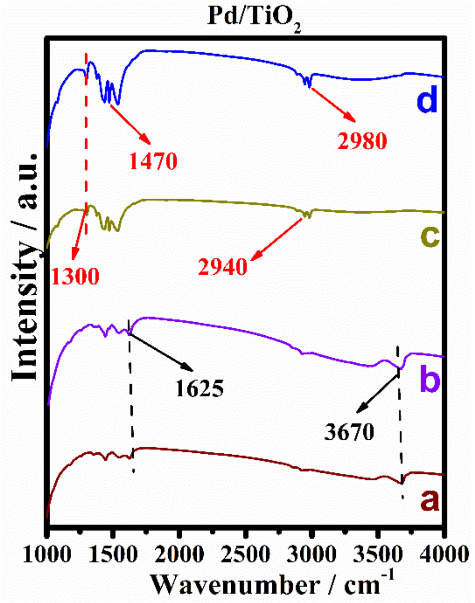

Figure S5. IR spectra of Pd/TiO_2 with propionic acid adsorbed for (a) shorter time and (b) longer time, and then irradiated with UV-Vis light for (c) 30 min and (d) 60 min.

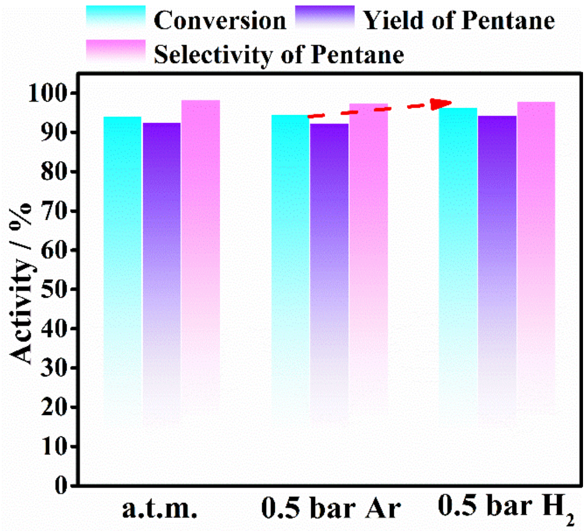

Figure S6. Effect of atmosphere on the photodecarboxylation of hexanoic acid over 1.5Au-0.8Pd/TiO_2 photocatalyst. Reaction conditions: 30 mg hexanoic acid, 10 mL dodecane, room temperature, 4 h.

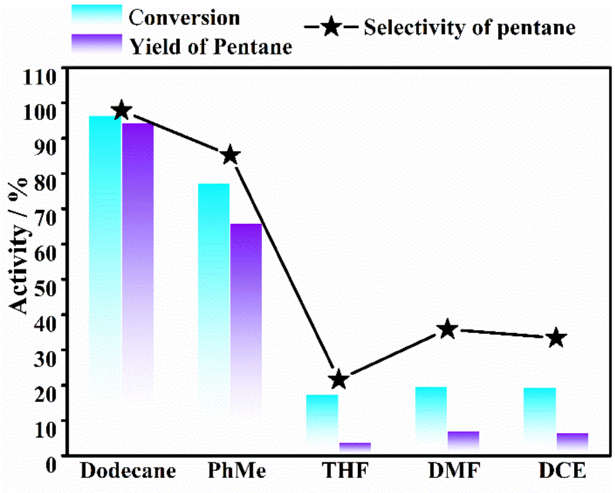

Figure S7. Effect of solvent on the photodecarboxylation of hexanoic acid over 1.5Au-0.8Pd/TiO_2 catalyst. Reaction conditions: 30 mg hexanoic acid, 10 mL solvent, room temperature, 4 h, 20 mg catalysts and 0.5 bar Ar.

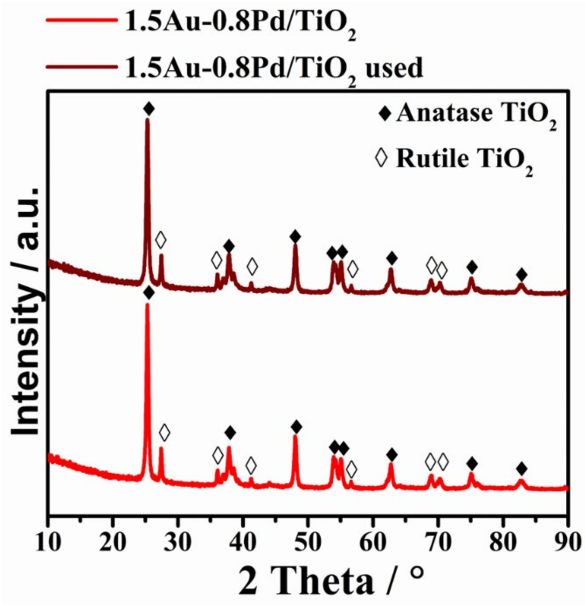

Figure S8. The XRD patterns of the fresh and used 1.5Au-0.8Pd/TiO_2 catalysts.

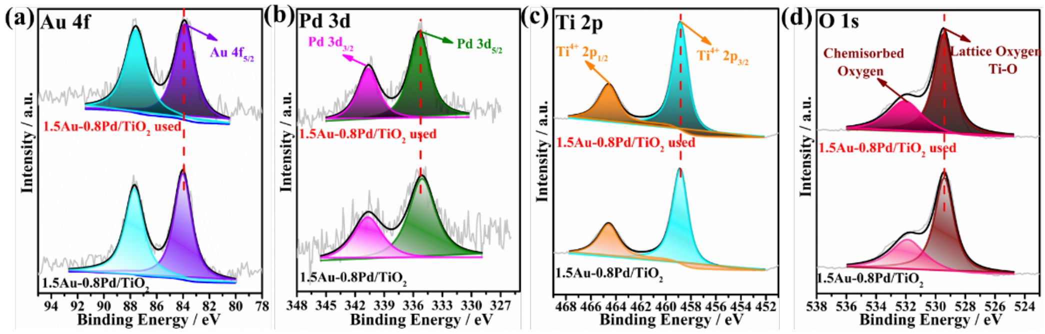

Figure S9. (a) Au 4f, (b) Pd 3d (c) Ti 2p and (d) O 1s XPS spectra of fresh and used 1.5Au-0.8Pd/TiO_2 catalysts.

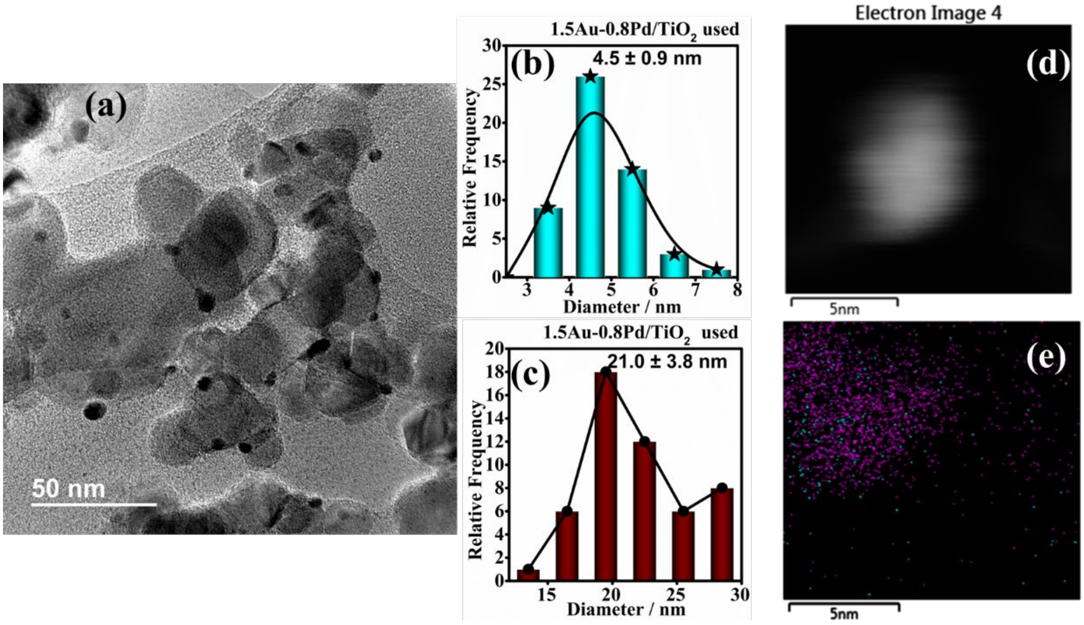

Figure S10. The (a) TEM image, (b) metal particle size distribution, (c) TiO_2 particle sizes distribution, (d) STEM image and (e) EDX-Mapping of used 1.5Au-0.8Pd/TiO_2 catalysts.

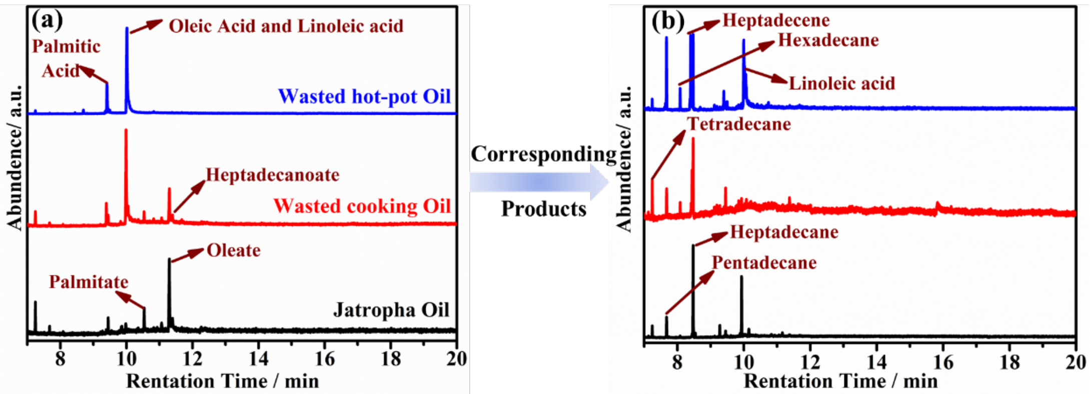

Figure S11. The GC-MS spectra of (a) crude oil including jatropha oil, wasted cooking oil and wasted hot-pot oil, (b) corresponding products by photocatalytic decarboxylation of these crude oils. Reaction conditions: 30 mg crude bio-oils, 10 mL dodecane, room temperature, 4 h, 20 mg catalysts, 0.5 bar H_2.

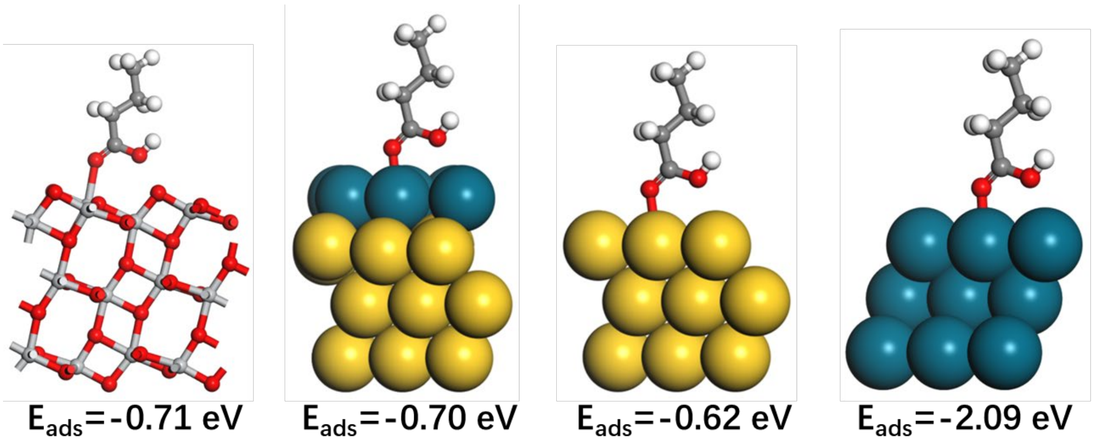

Figure S12. Optimized geometry models and corresponding adsorption energies of RCOOH adsorbed on TiO_2 (101) surface, Au@Pd model, Au and Pd model, respectively from left to right.

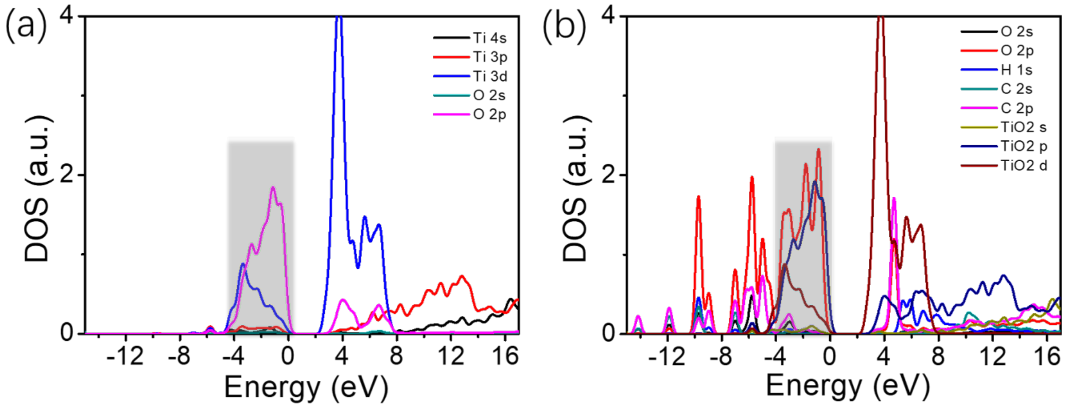

Figure S13. (a) PDOS of Ti and O orbitals in TiO_2 adsorbed sites. (b) PDOS of O, H, and C orbitals in RCOOH and TiO_2 orbital in TiO_2 adsorbed sites.

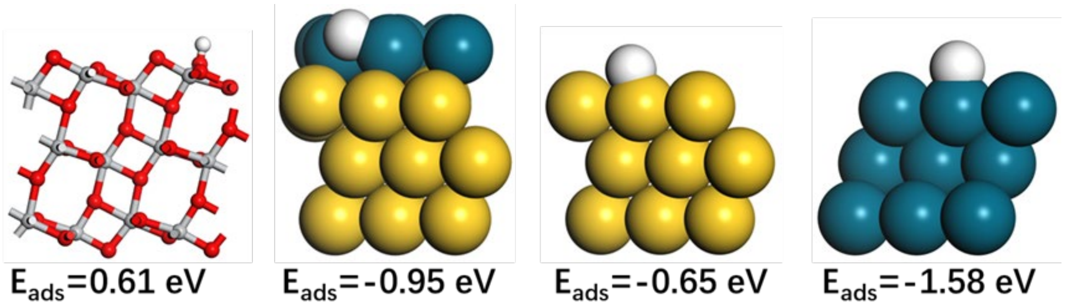

Figure S14. Optimized structural models and corresponding adsorption energies of H^+ adsorbed on TiO_2 (101) surface, Au@Pd model, Au and Pd model, respectively from left to right.

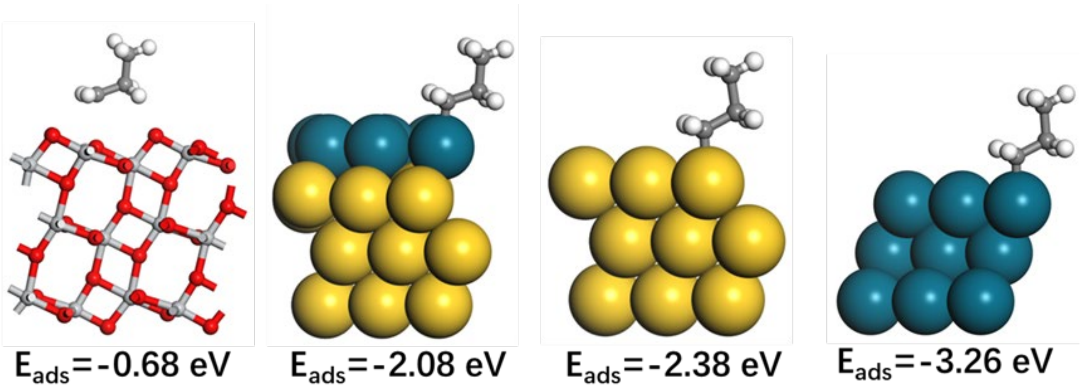

Figure S15. Optimized structural models and corresponding adsorption energies of R• adsorbed on TiO_2(101) surface, Au@Pd model, Au and Pd model, respectively from left to right.

Table S1. Theoretical and experimental loadings of Au and Pd on different catalysts.

Catalysts	Metal Loading (%)			
	Au		Pd	
	Theory	Actual	Theory	Actual
1.5Au/TiO_2	1.5	0.89	0	0
0.8Pd/TiO_2	0	0	0.8	0.65
1.5Au-0.8Pd/TiO_2	1.5	0.87	0.8	0.60
1.5Au-1.8Pd/TiO_2	1.5	0.72	1.8	1.53
1.5Au-5Pd/TiO_2	1.5	0.73	5	4.64

Table S2. Photocatalytic data at 2 h irradiation of octanoic acid photodecarboxylation in the presence of different catalysts.

Catalysts	Conversion (%)	Pentane Yield (%)	Pentane Selectivity (%)
P25 TiO_2	14.4	14.2	98.6
Au/TiO_2	70.0	66. 9	95. 6
Pd/TiO_2	89.5	85.1	95.1
1.5Au-0.8Pd/TiO_2	94.7	93.0	98.2
1.5Au0.8Pd alloy/TiO_2 [a]	51.0	42.3	82.9
Mixed 1.5Au/TiO_2 +0.8Pd/TiO_2 [a]	83.3	78.5	94.2

[a] These reactions were conducted for 11 h.

Table S3. Activity results for hexanoic acid conversion under different conditions.

Catalysts	Filter (nm)	Conversion (%)	Yield (%)	Selectivity (%)
No catalyst	No filter	0	0	0
1.5Au-0.8Pd/TiO_2	No filter	94.7	93.0	98.0
1.5Au-0.8Pd/TiO_2	360	71.2	68.8	96.4
1.5Au-0.8Pd/TiO_2	455	14.2	4.4	31.0
1.5Au-0.8Pd/TiO_2[a]	No filter	0	0	0

[a]The reaction was conducted under 100 ^{o}C in the dark.
